# Characterization of a novel organic solute transporter homologue from *Clonorchis sinensis*

**DOI:** 10.1371/journal.pntd.0006459

**Published:** 2018-04-27

**Authors:** Yanyan Lu, Won Gi Yoo, Fuhong Dai, Ji-Yun Lee, Jhang Ho Pak, Woon-Mok Sohn, Sung-Jong Hong

**Affiliations:** 1 Department of Medical Environmental Biology, Chung-Ang University College of Medicine, Seoul, Korea; 2 Department of Convergence Medicine University of Ulsan College of Medicine and Asan Institute for Life Sciences, Asan Medical Center, Seoul, Korea; 3 Department of Parasitology and Institute of Health Sciences, Gyeongsang National University School of Medicine, Jinju, Korea; Queen's University Belfast, UNITED KINGDOM

## Abstract

*Clonorchis sinensis* is a liver fluke that can dwell in the bile ducts of mammals. Bile acid transporters function to maintain the homeostasis of bile acids in *C*. *sinensis*, as they induce physiological changes or have harmful effects on *C*. *sinensis* survival. The organic solute transporter (OST) transports mainly bile acid and belongs to the SLC51 subfamily of solute carrier transporters. OST plays a critical role in the recirculation of bile acids in higher animals. In this study, we cloned full-length cDNA of the 480-amino acid OST from *C*. *sinensis *(CsOST). Genomic analysis revealed 11 exons and nine introns. The CsOST protein had a ‘Solute_trans_a’ domain with 67% homology to *Schistosoma japonicum* OST. For further analysis, the CsOST protein sequence was split into the ordered domain (CsOST-N) at the N-terminus and disordered domain (CsOST-C) at the C-terminus. The tertiary structure of each domain was built using a threading-based method and determined by manual comparison. In a phylogenetic tree, the CsOST-N domain belonged to the OSTα and CsOST-C to the OSTβ clade. These two domains were more highly conserved with the OST α- and β-subunits at the structure level than at sequence level. These findings suggested that CsOST comprised the OST α- and β-subunits. CsOST was localized in the oral and ventral suckers and in the mesenchymal tissues abundant around the intestine, vitelline glands, uterus, and testes. This study provides fundamental data for the further understanding of homologues in other flukes.

## Author summary

Clonorchiasis is a neglected tropical disease caused by the liver fluke, *Clonorchis sinensis*. *C*. *sinensis* is a biological carcinogen, causing cholangiocarcinoma in humans. Juvenile flukes migrate to and grow to adults in the bile ducts. The bile is chemo-attractive to the flukes and stimulates growth and egg production, and therefore is essential for their physiological and reproductive activities. However, excess bile accumulation can be toxic to the flukes. Several bile transporters are indispensable for *C*. *sinensis* to maintain homeostasis of bile acids in the bile duct. The *C*. *sinensis* organic solute transporter (CsOST) was presently cloned and characterized for the first time in trematodes. Interestingly, CsOST might include OST α- and β-subunits based on the positional sequence and overall structural conservation, although OSTβ was not reported from invertebrates. Our findings suggest that CsOST takes part in the export of bile acids and inhibitors. This study will stimulate further research on the functions of OST of liver flukes.

## Introduction

*Clonorchis sinensis* is a liver fluke that parasitizes the bile ducts of mammals [1]. Clonorchiasis was prevalent in China [2], Thailand [3], Vietnam [4], and Korea [5]. Globally, an estimated 15 million people are infected with *C*. *sinensis*, with most of these cases occurring in China [2]. Furthermore, *C*. *sinensis* causes numerous pathophysiological primary and secondary changes, such as inflammation, hyperplasia of epithelial cells, metaplasia in the mucosa, and periductal fibrosis [6]. The World Health Organization has listed clonorchiasis as a Group 1 biological carcinogenic agent that causes cholangiocarcinoma in humans[7].

Bile is a substantially toxic solution even under normal conditions [8]. Bile toxicity can be a primary consequence of atypical bile composition. The lithocholic acid (LCA) component of bile acid is very hydrophobic and toxic. Feeding of LCA intrinsically causes bile duct injury in mice [9]. Furthermore, insufficient dilution and rinsing of bile contributes to the alkalinity and hydration of bile, which eventually damages the bile duct [10]. In addition to abnormal bile composition, disturbances of either normal flow or transport of bile across membranes may also result in the formation of toxic bile. Bile toxicity can be prevented by its export via bile transporters. These transporters include multidrug resistance proteins 3 (MRP3) [11], MRP4 [12], bile salts export pump (BSEP) [13], and organic solute transporter (OST) [14]. Bile flow and circulation via the bile efflux transporters is crucial in reducing bile toxicity and limiting its interaction with cell membranes.

Bile acid transporters are crucial for the survival of *C*. *sinensis* since bile acids could be toxic to this fluke. Bile content greater than 0.1% is lethal to *C*. *sinensis* juveniles and LCA is toxic to the worms [15]. *C*. *sinensis* transporters that reduce bile acids in the fluke [16] may include OST, MRP3, MRP4, and BSEP.

Among the bile acid transporters, OST is an exporter which disposes of bile acids, steroid conjugates, and prescription drugs [13]. The OST species, designated as SLC51, belongs to the solute carrier (SLC) superfamily, which comprises 52 families. The members of this family transport a broad spectrum of substrates including nutrients, toxins, and drugs [17]. OSTα (SLC51A) and OSTβ (SLC51B) subunits form a heterodimer and together function as a transporter [18]. Human OSTα (HsOSTα) shares high amino acid identity (83%) with mouse OSTα (MmOSTα), but moderate identity (41%) with OSTα of little skate (*Leucoraja erinacea*) (LeOSTα). HsOSTβ also displays moderately high identity (63%) with mouse OSTβ (MmOSTβ) but low identity (25%) with *L*. *erinacea* OSTβ (LeOSTβ) [19]. Regardless of the low sequence identity between human and skate, heterodimerization of the α and β subunits from different species may mediate transport activity, suggesting evolutionary functional conservation across species [20].

Here, we report a molecular, biochemical, and computational structural characterization of *C*. *sinensis* OST (CsOST). This is the first report of a trematode-specific OST. The structure of CsOST was predicted and investigated in detail using rational three-dimensional (3D) modeling methods. The CsOST was localized in adult *C*. *sinensis*.

## Materials and methods

### Ethics statement

Animal experiments were approved by the Institutional Animal Care and Use Committee at Chung-Ang University (Approval Number 2015–00005). This study was carried out in strict accordance with the national guidelines outlined by the Korean Laboratory Animal Act (No. KCDC-122-14-2A) of the Korean Centers for Disease Control and Prevention (KCDC).

### Parasites and animals

Fresh-water fish *Pseudorasbora parva* (Jinju, Korea) were digested with artificial gastric juice at a ratio of 1:10 as described previously [21]. The slurry was filtered through a 1-mm diameter sieve to remove debris and the filtered *C*. *sinensis* metacercariae that settled to the bottom of the beaker were washed several times with 0.85% saline. The metacercariae were collected under a dissection microscope and stored in sterilized phosphate-buffered saline (PBS) containing antibiotics (Antibiotic-Antimycotic, 100× liquid; Gibco, Grand Island, NY, USA) at 4°C.

New Zealand White rabbits (2.2–2.4 kg; Koatech, Gyeonggi-do, Korea) were each infected orally with 250 metacercariae twice using a gastric tube, with an interval of 1 week between the oral doses. After 1 month, *C*. *sinensis* adult worms were recovered from the bile ducts of killed rabbits. The flukes were washed several times with sterilized PBS.

### Synthesis of CsOST cDNA

Total RNA was extracted using TRIzol reagent (Invitrogen, Carlsbad, CA, USA) according to the manufacturer’s instructions and treated with RNase-free DNase using a DNA-free kit (Ambion, Austin, TX, USA) to remove the trace genomic DNA [22]. The quality of the total RNA was validated by the ratio of the optical density determined at 260 and 280 nm using a NanoDrop 1000 Spectrophotometer (Thermo Scientific, Waltham, MA, USA). First-strand cDNA was synthesized using the power cDNA Synthesis kit with the Oligo (dT)_15_ primer (INtRON Biotechnology, Seongnam, Korea) for quantitative real-time PCR (qRT-PCR) or using the SMARTer RACE cDNA Amplification Kit (Clontech Laboratories, Inc., Mountain View, CA, USA) for rapid amplification of cDNA ends (RACE) according the manufacturer’s instruction. Synthesized total cDNA templates were stored at −20°C until analysis.

### Validation of full-length cDNA

Expressed sequence tag (EST) clone CSA09963, which encoded a homologous OST polypeptide, was retrieved from the *C*. *sinensis* transcriptome database of the Korea National Institute of Health [23, 24]. Plasmid DNA was extracted from its glycerol stock and sequenced (Macrogen, Inc., Seoul, Korea). The missing N-terminal region was obtained using 5′-RACE (SMARTer RACE cDNA Amplification Kit; Clontech Laboratories, Inc.). A gene-specific primer (5′–CTGATACATCCCACATCGACCACCAAGACG–3′) and nested gene-specific primer (5′–TGGTGAGCGAGGGCGGCGAAGAACATCTC–3′) were designed and synthesized (Bioneer, Daejeon, Korea). The cDNA amplification conditions were as follows: pre-denaturation (94°C for 5 min), amplification (35 cycles of 94°C for 30 s, 60°C for 30 s, 72°C for 1 min), and final extension (72°C for 10 min). The PCR product was purified using the QIAquick PCR purification kit (QIAGEN, Hilden, Germany) and subcloned into the pCR2.1-TOPO vector using a TOPO TA cloning kit (Invitrogen). After re-confirming the results by colony PCR and restriction enzyme digestion, the plasmid DNA was sequenced (Macrogen, Inc.). Finally, the sequences obtained from the CSA09963 clone and through 5′-RACE were combined for the intact cDNA of CsOST.

To validate the ligated full-length product, two primers were designed at the two ends and PCR was performed on total cDNA of *C*. *sinensis*. The forward primer was 5′–TGCTTTACCAGCTGAGTCTGTGGCTTTCGA–3′ and the reverse primer was 5′–AAGCCATCCAGCCACGAACAGCCTATAGA–3′. PCR conditions were the same as detailed above. The PCR product was purified and applied for TA cloning as described, and then confirmed by sequencing.

### Features of genomic and amino acid sequence

*CsOST*-related genomic DNA sequences were found in a genome assembly of *C_sinensis*-2.0 (Assembly ID: GCA_000236345.1) by a BLASTN search with the CsOST cDNA sequence. At the genome level, two *C*. *sinensis* DNA scaffolds on CsOST were also found by BLASTN searching and compared with the CsOST cDNA sequence. The genomic organization map was constructed based on manual inspection and comparison.

A putative CsOST polypeptide was deduced from the CsOST cDNA sequence using the EMBOSS Sixpack software (http://www.ebi.ac.uk/Tools/st/emboss_sixpack/). The N-terminal signal peptide was predicted using SignalP 4.1 [25]. CsOST was recognized by BLASTP [26] in the NCBI non-redundant (NR) database, and its information was confirmed using the UniProtKB/Swiss-Prot database [27]. Functional and conserved domains were searched using the InterProScan [28] and NCBI Conserved Domains database (CDD) [29]. Gene Ontology (GO) for specific biological process, molecular function, and cellular component were assigned using AmiGO ver. 2.4.26 [30].

### Multiple sequence alignment and phylogenetic analysis

The amino acid sequences of OST subunits were retrieved from the UniProtKB/Swiss-Prot database [27]. Multiple sequence alignments were carried out with the parameters of G-INS-i method, unalign level of 0.8, and BLOSUM30 scoring matrix using MAFFT ver. 7.394 [31], and were visualized using Jalview [32]. A phylogenetic tree of homologous proteins was constructed using maximum parsimony methods of MEGA ver. 6.06 [33]. The probability of clustering with taxa was calculated using bootstrap test with 1000 replicates.

### CsOST-N and -C for building the tertiary model

CsOST was split into two domains, comprising an N-terminal ordered (or folded) domain (CsOST-N; Met1 to Arg354; 354 aa) and C-terminal disordered (or unfolded) domain (CsOST-C; Arg355–Leu480; 126 aa) using DISOPRED3 [34]. To build a molecular model, a short fragment (Met1–Leu23) was removed, and the remainder of CsOST-N and CsOST-C were used for accurate model prediction. Initial three-dimensional (3D) models of CsOST-N and -C were generated using the Local meta-threading-server (LOMETS) [35]. The top 10 models were ranked in order of decreasing confidence score. All models were refined by solvation and energy minimization using YASARA [36]. Potential error in the 3D model was evaluated using a Ramachandran plot [37] and ERRAT [38], which were implemented in the Structure Analysis and Verification Server (http://services.mbi.ucla.edu/SAVES/). Other OST subunits were also modeled using the same methods. Overall structural comparisons were performed using the TM-align structure alignment server [39]. The TM-score value indicates the overall fold similarity. It is protein size-independent and less sensitive to local structural variations. TM-score ranges from 0 to 1, where 1 indicates 100% similarity between two structures. Thus, the value was displayed as a percent value to simplify the analysis. Finally, we selected the most evolutionarily conserved structure of each OST subunit, performing pairwise comparisons of the top 10 3D models obtained from LOMETS between different species. Structure visualization was carried out using UCSF CHIMERA [40].

### Quantitative real-time PCR

Total RNA samples of the adults and metacercariae of *C*. *sinensis* were prepared in triplicate. A relative mRNA expression level of CsOST in different developmental stages was measured by qRT-PCR using LightCycler FastStart DNA Master SYBR Green I (Roche, Basel, Switzerland), with 50 ng total cDNA/reaction. To carry out qRT-PCR, primers were designed utilizing Oligo-primer analysis software ver. 6.71 (Molecular Biology Insights, Cascade, WA, USA). The forward primer was 5′-CACAGTGATACGACCGATAAC-3′ and the reverse primer was 5′-CGAACAAGTCCCGACGCATA-3′. Phosphoglycerate kinase, which is expressed stably in developmental stages of *C*. *sinensis*, was employed as a reference gene [41]. qRT-PCR was conducted in LightCycler2.0 and the relative expression level of CsOST in developmental stages was calculated using 2^-ΔΔCt^ equation [42].

### Recombinant CsOST fragment production

To produce soluble recombinant protein with high antigenicity, the full-length amino acid sequence of CsOST was analyzed with Antibody Epitope Prediction (http://tools.immuneepitope.org/bcell/) and hydrophilicity prediction using ProtScale (http://web.expasy.org/protscale/). Regions with high immunogenicity and high hydrophilicity were chosen for recombinant protein production. The target region was amplified by PCR using forward primer (5′-GATATGGATCCCTCGGTCCCAATCGCCGTC–3′) and reverse primer (5′–CTGCGAAGCTTTAGAGTGACAAACGGATCTTGG–3′). The PCR template was CsOST full-length cDNA. The PCR product was purified with QIAquick PCR purification kit (QIAGEN) and subcloned into the pET-23a(+) vector (Novagen, Darmstadt, Germany) containing a 6× histidine tag.

After transformation into BL21(DE3)pLysS competent cells (Promega, Fitchburg, WI, USA), a single colony was picked up and cultured into LB broth overnight. Recombinant protein was induced by adding 10 μM of isopropyl β-D-thiogalactopyranoside at 30°C overnight. The bacteria were harvested by centrifugation at 4000 rpm for 10 min, resuspended in lysis buffer (1×PBS with 1 mg/mL lysozyme, 1 mM phenylmethylsulfonyl fluoride, 1 mM dithiothreitol, 1 μg/mL leupeptin, and 1 μg/mL pepstatin), and sonicated using a QSONICA Q700 apparatus (QSONICA, Newtown, CT, USA). After centrifugation, the target protein in the supernatant was purified by binding to Ni-NTA agarose (QIAGEN) and washing with 1×PBS containing 20 mM imidazole. The His-tagged recombinant CsOST (rCsOST) protein was eluted with elution buffer I (1× PBS and 100 mM imidazole) followed by three volumes of elution buffer II (1×PBS and 200 mM imidazole). The purified protein was confirmed by resolution on a 12% gradient gel (ELPIS, Daejeon, Korea) and western blotting. The primary antibody was mouse anti-Penta His antibody (1:1000; Thermo Scientific, Waltham, MA, USA) and the secondary antibody was goat AP-conjugated anti-mouse IgG antibody (1:5000; Sigma-Aldrich, St. Louis, MO, USA). The target protein was detected by color developed using 5-bromo-4-chloro-3-indoyl phosphate/nitroblue tetrazolium (Invitrogen) as the substrate.

### Mouse immune sera

Mice non-specifically reactive to *C*. *sinensis* proteins were strictly excluded prior to immunization. Sera was obtained from the mice from tail vein and blotted against the soluble extract of *C*. *sinensis* by immuno-enhanced chemiluminescence (ECL) using BS ECL Plus kit (Biosesang, Seongnam, Korea). Mice that were non-reactive to *C*. *sinensis* were selected for immunization.

To increase the specific response of the target antibody, the Ni-NTA eluate was run on a 12% gradient gel, the rCsOST band was cut out, and was equilibrated in sterile 1×PBS at 4°C. The gel slice containing the rCsOST band was thoroughly homogenized using a Pyrex Glass Pestle Tissue Grinder (Corning, Inc., Corning, NY, USA) in 1×PBS on ice. The liquid homogenate was injected into the peritoneum of female BALB/c mice (8 weeks old; Orient Bio, Seongnam, Korea). Two weeks later, the same amount of the rCsOST homogenate was injected intraperitoneally into the other quadrant of the mice. After 2 weeks, blood was collected from the eye veins of the immunized mice. Sera were stored at –20°C and used as the primary antibody to detect native CsOST.

### Detection of native CsOST in *C*. *sinensis*

After recovery from the rabbit liver, the *C*. *sinensis* adult flukes were washed with pre-cooled sterile 1×PBS. The cytosolic and membrane fractions were extracted using the Mem-PER Plus Membrane Protein Extraction Kit (Thermo Scientific) following the manufacturer’s instructions. The protein concentration of cytosolic and membrane fractions was determined using a protein assay kit (Bio-Rad, Hercules, CA, USA). The fractions were dispensed as aliquots and stored at –70°C until use.

The cytosolic and membrane fractions (100 μg/well) and rCsOST were resolved on a 12% gradient gel and transferred to a nitrocellulose membrane. The membrane was blocked with 5% skim milk for 1 hr at room temperature and incubated in mouse anti-CsOST serum (1:200) overnight at 4°C. After washing three times with PBS-Tween 20, the membrane was incubated in goat alkaline phosphatase-conjugated anti-mouse IgG at 1:10000 dilution (Sigma-Aldrich) at room temperature for 3 hr. After washed three times with PBS-Tween 20 and ECL was carried out using the BS ECL Plus kit (Biosesang, Seongnam, Korea). Target bands were detected using ImageQuant LAS 4000 (GE Healthcare Life Sciences, Little Chalfont, UK).

### Immunohistochemical staining

For immunohistochemical staining, paraffin ribbons of *C*. *sinensis*-infected rabbit liver were prepared and processed as described previously [21]. The ribbons were incubated with mouse anti-CsOST immune serum or normal mouse serum, which were diluted at 1:100 for the adults and 1:400 for the metacercariae with antibody diluent solution (Life Science Division, Mukilteo, WA, USA) at 4°C overnight. The ribbons were incubated in Dako EnVision+System–horseradish peroxidase–labelled polymer anti-mouse IgG (Dako Cytomation, Glostrup, Denmark) diluted 1:400 at room temperature for 1 hr and rinsed with TBS.

## Results

### Full-length sequence of CsOST

The CsOST EST clone (CSA09963) was 732 base pairs (bp) in length and contained a 3′-poly(A) tail. The 5′-end sequence of CsOST was obtained by 5′-RACE (S1 Fig). The RACE PCR-amplified product was estimated to be 1200 bp by primary PCR and was confirmed as 1100 bp by nested PCR generating amplicon (S2 Fig). When sequences of the EST clone and RACE product were assembled, the full-length cDNA of CsOST was elongated to 1725 bp, encoding a putative polypeptide of 480 amino acids and containing 5′-untranslated region (5′-UTR; 143 bp) and 3′-UTR (139 bp) (S1 Fig). The full-length cDNA and putative polypeptide sequences of the CsOST were determined (S3 Fig).

### Genomic organization of *CsOST*

**Fig 1:**
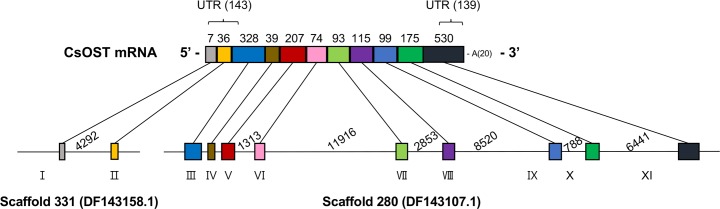
Schematic representation of the genomic structure of *CsOST*. 5′ UTR (143 bp) located at exon I, exon II, and a part of exon III (100 bp), while 3′-UTR was located in the posterior region including exon XI (117 bp). The poly(A)-tail was 22 bp in length. The lengths of intron (shown as a solid line) and exon (shown as a colored box) are proportional to each size.

Genomic organization analysis showed that *CsOST* mRNA consisted of 11 exons edited by *cis*- and *trans*-splicing. Two exons at the anterior end were derived from *C*. *sinensis* DNA scaffold 331 (GenBank ID: DF143158.1) and the remaining nine exons were embedded in *C*. *sinensis* DNA scaffold 280 (GenBank ID: DF143107.1). By *trans*-splicing, the anterior and posterior premature RNAs were connected to a mature mRNA. The 5′-UTR of *CsOST* mRNA consisted of exon I (7 bp), exon II (36 bp), and partial exon III (100 bp). The *CsOST* open reading frame (ORF) spanned from exon III (remaining 228 bp) to exon XI (anterior 413 bp). The 3′-UTR was an anterior part of exon XI (posterior 117 bp) (Fig 1).

### Functional annotation and motifs of CsOST

The deduced CsOST polypeptide was most similar, with 67.0% identity, to a putative OST of *Schistosoma mansoni* (GenBank ID: XP_018651812.1) in the NCBI NR database. The CsOST was annotated based on the 23.1% similarity to OST α-subunit of *Xenopus tropicalis* (XtOSTα; UniProt ID: A9ULC7) with an E-value 4.9e-11 in the UniProtKB/Swiss-Prot database [27], which consists of a high-quality, manually annotated, and reviewed data. A functional domain search against the CDD revealed a ‘Solute_trans_a’ (Pfam ID: PF03619) in the internal region (Phe42 to Pro308) of the CsOST, which belongs to ‘organic solute transporter subunit alpha/Transmembrane protein 184’ (OSTα/TMEM184C, IPR005178). The CsOST did not have a signal peptide. It was assigned to GO terms integral component of membrane (cellular component, GO:0016021), transport (biological process, GO:0006810), and transporter activity (molecular function, GO:00005215).

### Sequence features of CsOST-N and -C termini as OSTα- and β-subunits

**Fig 2:**
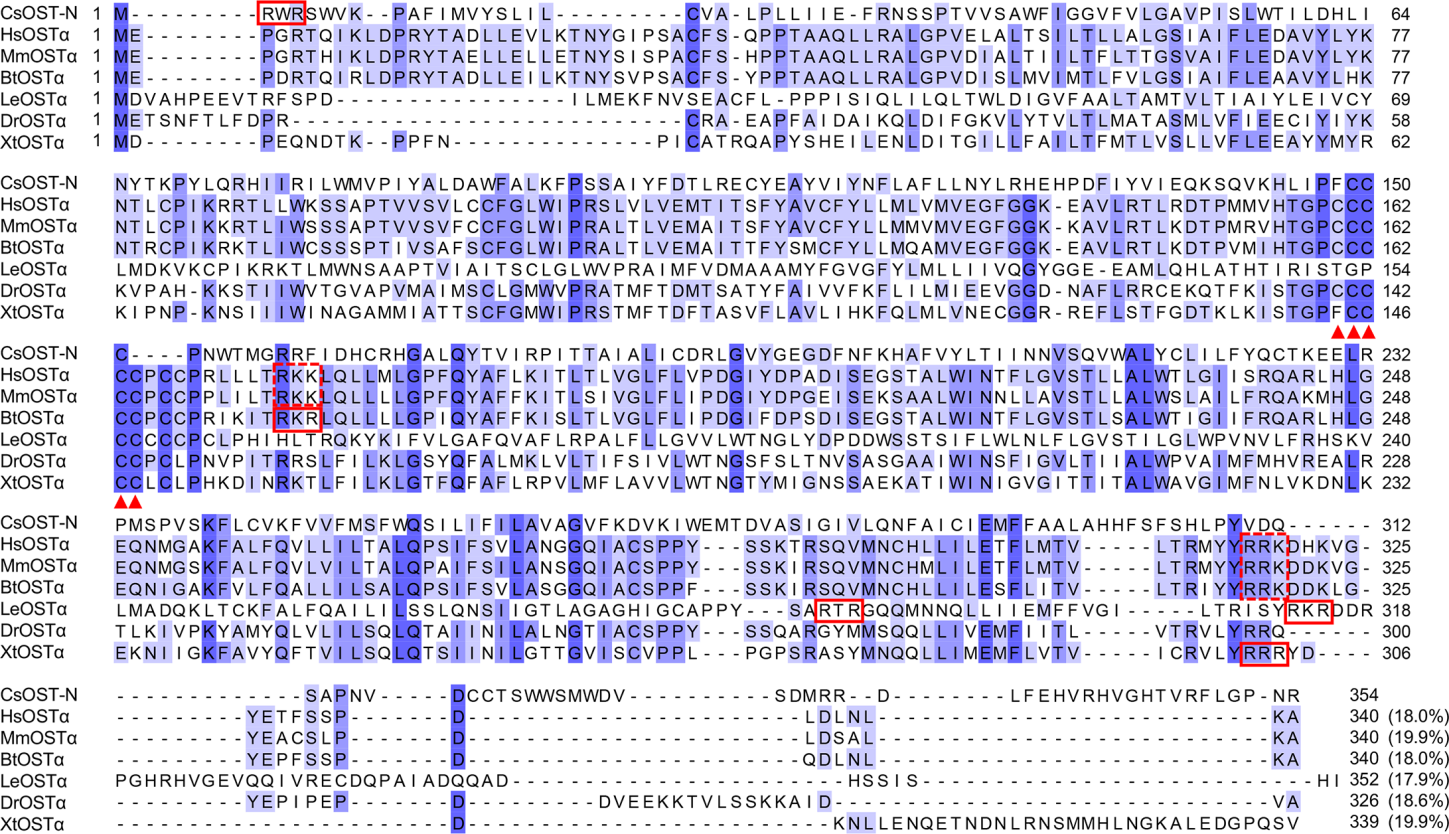
Sequence conservation of CsOST-N and other OST α-subunits. CsOST-N is an N-terminal domain (Met1–Arg354) of CsOST. Residues that are 75% conserved among the alignment are shaded in *light blue* and those that are 100% conserved are highlighted in *dark blue*. The five consecutive cysteine residues are indicated with *red arrowheads*. The *solid red boxes* locate Arg-X-Arg (RXR) motif, and the *dotted red boxes* show Arg-Arg-Lysine (RRK) and Arg-Lys-Lys (RKK). Parentheses show pairwise sequence identity between CsOST-N and other OSTα subunits. Abbreviations are: CsOST-N, N-terminal regions of CsOST; HsOSTα, *Homo sapiens* OSTα; MmOSTα, *Mus musculus* OSTα; BtOSTα, *Bos taurus* OSTα; LeOSTα, *Leucoraja erinacea* OSTα; DrOSTα, *Drosophila melanogaster* OSTα; XtOSTα, and *Xenopus tropicalis* OSTα.

Four OSTα subunits from the vertebrate animals were highly conserved with a range of 34.7–82.7%, but CsOST-N showed relatively low conservation with a range of 16.6–20%. In OSTα of the vertebrate animals, a motif of five cysteine residues was highly conserved, whereas CsOST-N had three conserved cysteine residues (Fig 2). The Arg-X-Arg (RXR) motif, such as RWR, RKR and RRR, was found in CsOST-N, OSTα of *Bos taurus* (BtOSTα), *L. erinacea* (LeOSTα), and *X. tropicalis* (XtOSTα). The Arg-Arg-Lys (RRK) motif is found in mammals, while the Arg-Lys-Lys (RKK) motif was conserved in HsOSTα and MmOSTα.

**Fig 3:**
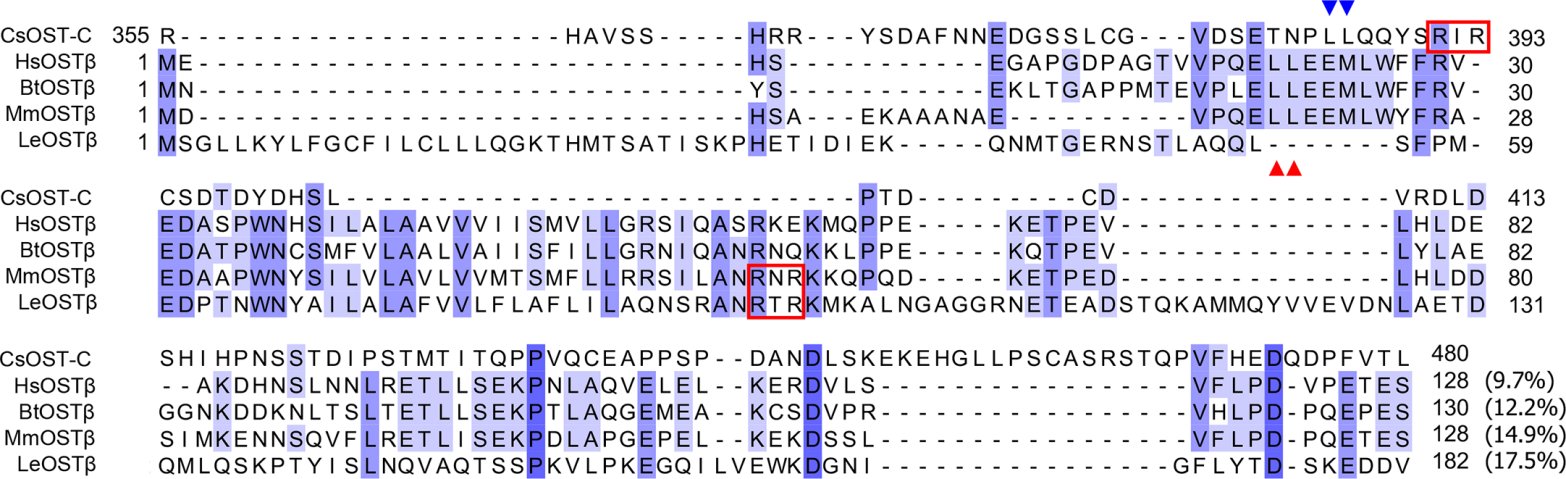
Comparison of the amino acid sequence of CsOST-C with OST β-subunits. CsOST-C is a C-terminal domain (Arg355–Leu480) of the CsOST. Conservation is the same as depicted in Fig 2. The di-leucine (LL) residues are labeled with *red* and *blue triangles*. The *solid boxes* in *red* indicate an Arg-X-Arg (RXR) motif, such as RIR, RNR, and RTR. Parentheses show pairwise sequence identities between CsOST-C and other OSTβ subunits. Abbreviations are: CsOST-C, C-terminal regions of CsOST; HsOSTβ, *H*. *sapiens* OSTβ; MmOSTβ, *M*. *musculus* OSTβ; LeOSTβ, *L*. *erinacea* OSTβ; BtOSTβ, and *B*. *taurus* OSTβ.

Like OST α-subunits, the RXR motifs were found in three OSTβ as RIR in CsOST-C, RNR in MmOSTβ, and RTR in LeOSTβ (Fig 3). A motif of di-leucine residues (LL) were identified in OSTβ of mammals and in CsOST-C (386 and 387 aa).

**Fig 4:**
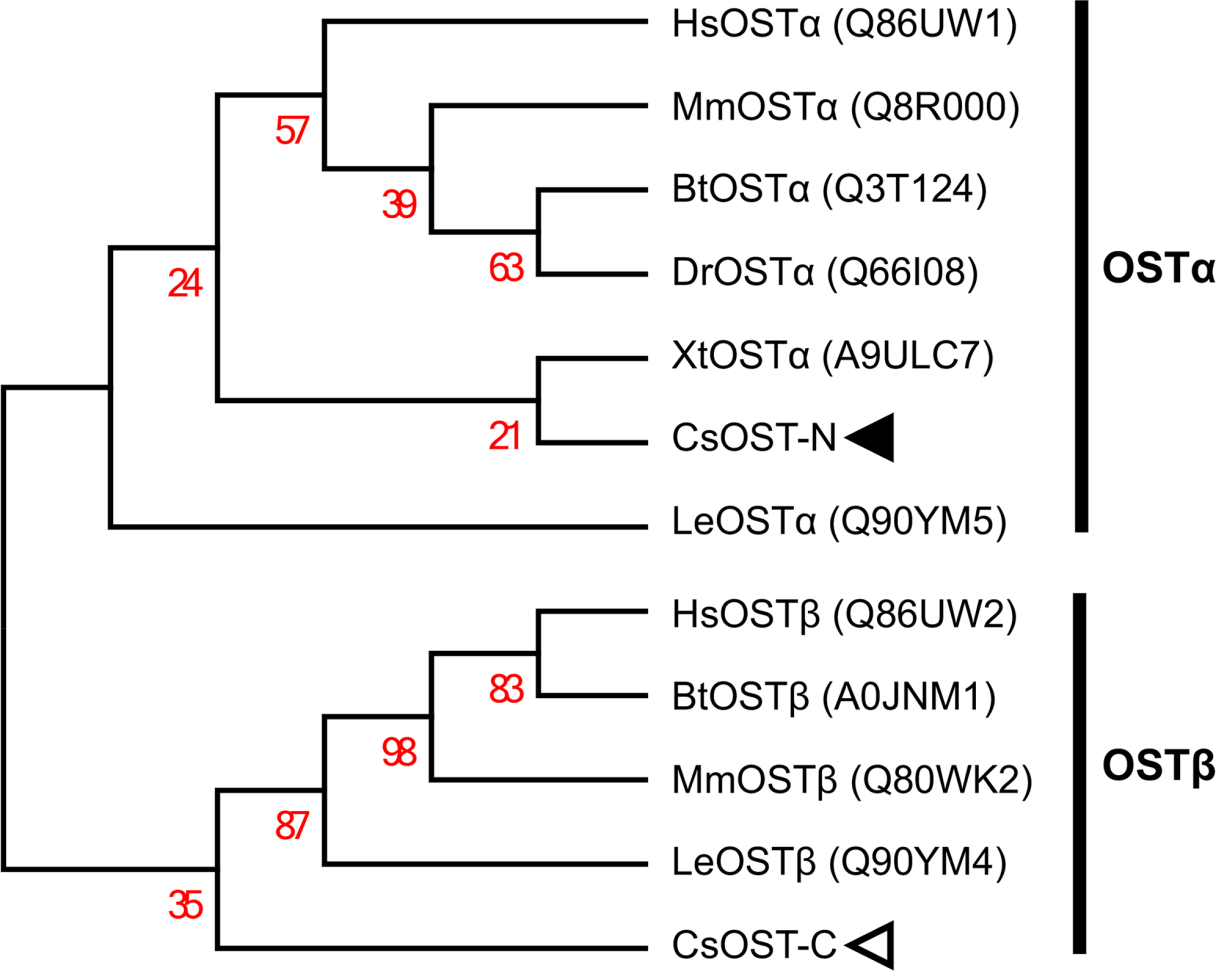
Phylogenetic tree of CsOST-N and -C domains with canonical OSTα and β subunits. Bootstrap values (1000 replicates) are shown next to the branches. UniProtKB/Swiss-Prot IDs [27] are shown in parentheses. CsOST-N and -C were indicated with closed and open arrowhead, respectively. Abbreviations are defined as in the legends of Figs 2 and 3.

The phylogenetic tree revealed that CsOST-N and -C were grouped into OSTα and OSTβ, respectively (Fig 4). CsOST-N was closely related to XtOSTα, supporting functional annotation by *X*. *tropicalis* with the significance value.

### Threading-based modeling and validation

The top 10 3D models of CsOST-N and -C domains were built based on multiple templates using LOMETS [35] and refined using YASARA energy minimization [36]. A final 3D model of each CsOST domain was determined according to stereochemical quality assessment as well as high structural similarity indicated as the TM-score on structural pairwise comparisons across species. The reason is that OST α/β-subunits, derived from different species, function complementarily to each other [20].

**Fig 5:**
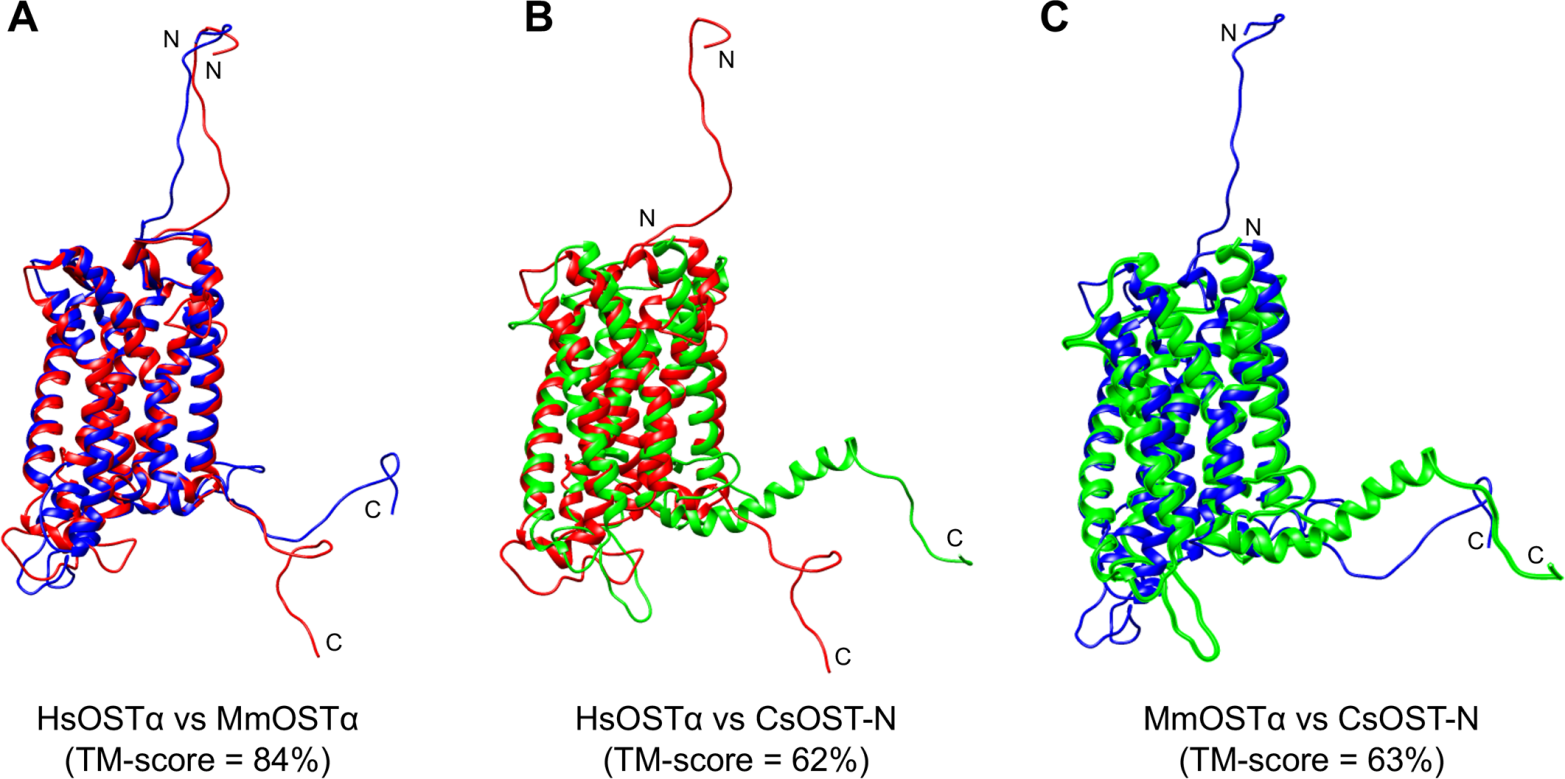
Structural comparison of CsOST-N with OSTα subunits. The 3D models of HsOSTα (*red*), MmOSTα (*blue*), and CsOST-N (*green*) are superimposed. Pairwise comparisons were performed with HsOSTα and MmOSTα (A), HsOSTα and CsOST-N (B), MmOSTα and CsOST-N (C). TM-score between the two superposed structures was calculated using TM-align [39]. Abbreviations are provided in the legend to Fig 2.

**Fig 6:**
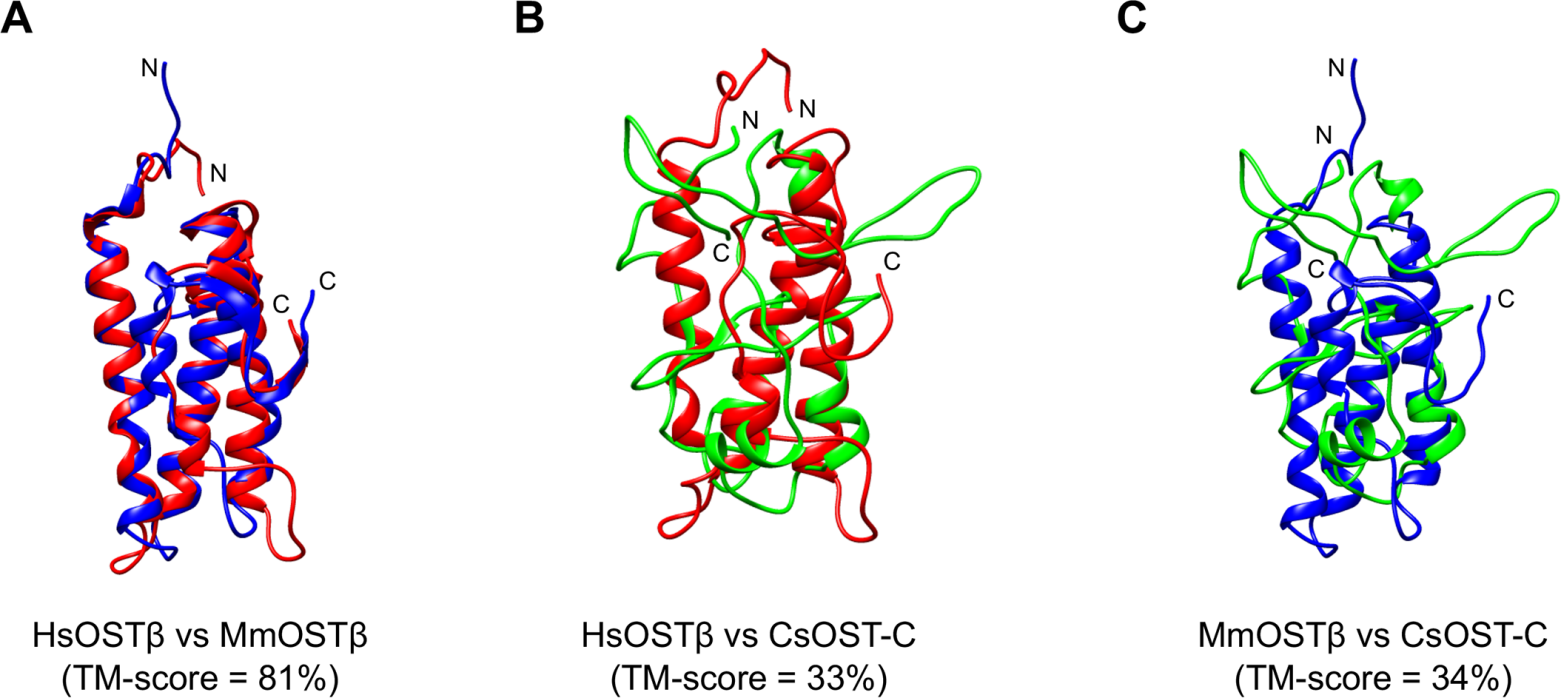
Structural comparison of the CsOST-C with OST β-subunits. The 3D models of HsOSTβ (*red*), MmOSTβ (*blue*) and CsOST-N (*green*) were superposed on each other. Pairwise comparisons were performed with as follows: HsOSTβ and MmOSTβ (A), HsOSTβ and CsOST-C (B) and MmOSTβ and CsOST-C (C). TM-score between two superposed structures was calculated using TM-align [39]. Abbreviations are provided in the legend to Fig 3.

For this purpose, 3D models of each subunit of HsOST and MmOST were also prepared as described above. All 3D models were compared between the HsOST and MmOST subunits to determine an evolutionarily conserved 3D model. HsOSTα (model no. 2) and HsOSTβ (model no. 5) were most similar to MmOSTα (model no. 2) and MmOSTβ (model no. 4), showing 85% and 83% TM-scores, respectively (Figs 5A and 6A; S1 and S2 Tables). Two OSTα models of HsOSTα and MmOSTα were applied to determine the most suitable model of CsOST-N. Model no. 1 of CsOST-N showed the highest similarity to the mammalian OSTα models, with TM-scores of 62% and 63% (Fig 5B and 5C; S3 Table). Two OSTβ models of HsOSTβ and MmOSTβ were used to determine the most acceptable model of CsOST-C. The most acceptable model of CsOST-C (model no. 4) was selected, with TM-scores of 33% and 34% against HsOSTβ and MmOSTβ, respectively (Fig 6B and 6C; S4 Table). In case of LeOSTβ, model no. 4 was selected. The model displayed the highest similarity (42% and 41%) to HsOSTβ and MmOSTβ, respectively (S4A and S4B Fig; S5 Table).

The final 3D model of CsOST-N was evaluated as a good model as follows. The Ramachandran plot verified the quality of the model, showing that 87.5% of residues were in the favorable region, 11.2% in the additionally allowed region, and only 0.7% of the residues (Cys141 and Glu170) in the generously allowed region. Only 0.7% of residues (Ile77 and Ala178) were in the disallowed region (S5A Fig). The overall quality score of 84.1% from ERRAT supported the accuracy of the 3D model (S5B Fig). The final 3D model of the CsOST-C domain revealed that 78.0% of residues were in the favorable region and 0.9% (Ser443) in the disallowed region in the Ramachandran plot with an ERRAT value of 77.1% (S6A and S6B Fig).

### Developmental expression and tissue distribution

**Fig 7:**
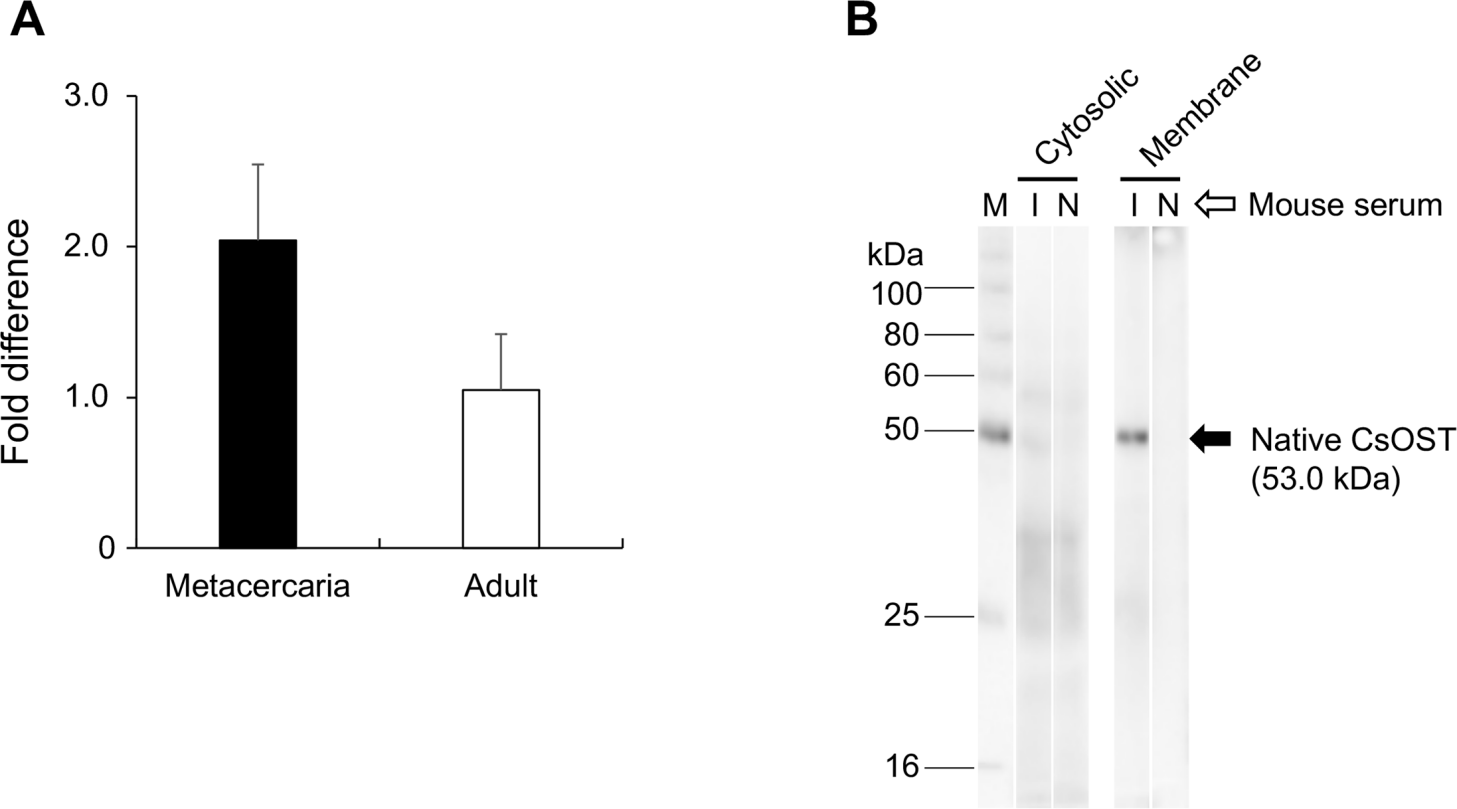
Relative mRNA level of CsOST and reactivity of mouse anti-CsOST-C immune serum. (A) CsOST was expressed 2-fold more in the metacercaria than in the adult. (B) Mouse immune serum reacted specifically with native CsOST in the cytosolic and membrane fractions of *C*. *sinensis*. Abbreviations are: *M*, protein molecular marker; *I*, immune mouse serum; and *N*, normal mouse serum.

**Fig 8:**
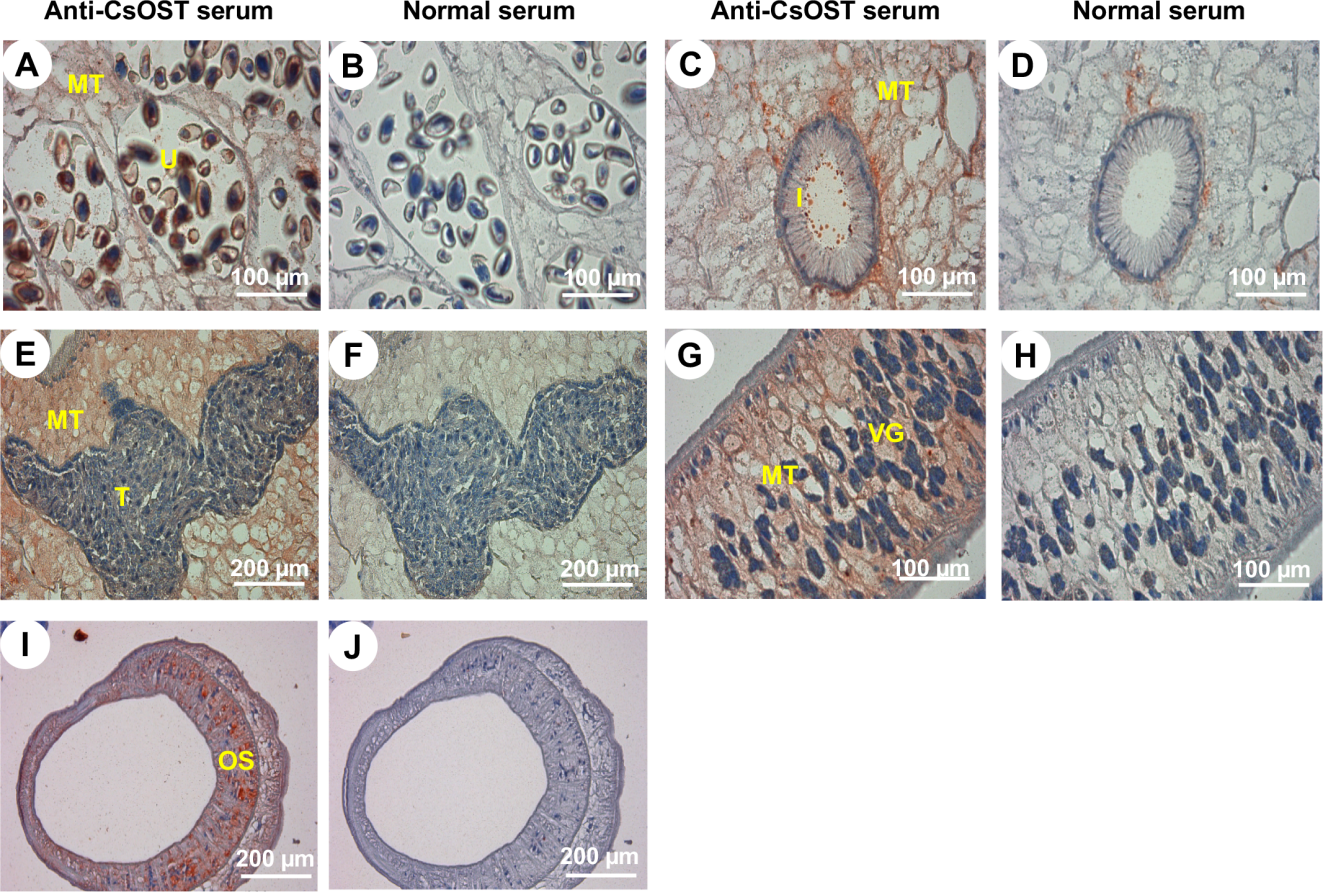
Distribution of CsOST in *C*. *sinensis* adults. Panels A, C, E, G, and I display samples treated with mouse anti-CsOST serum at 1:100 dilution. Panels B, D, F, H, and J display samples incubated with normal mouse serum. Abbreviations are: *I*, intestine; *MT*, mesenchymal tissue; *OS*, oral sucker; *T*, testis; *U*, uterus; and *VG*, vitelline gland.

CsOST mRNA was detected both from the adults and metacercariae of *C*. *sinensis* by qRT-PCR. CsOST mRNA was 2-fold more abundant in the metacercariae than in the adults (Fig 7A). A region (Arg348–Leu480) employed for the production of the recombinant CsOST protein was determined based on both B-cell epitope and hydrophilicity predictions (S7 Fig). This approach has helped to effectively produce and purify an antigenic fragment of a novel protein, such as membrane-spanning transporter MRP7 of *C*. *sinensis* (CsMRP7) and Parkin [21, 22]. Through Ni-NTA purification, a rCsOST fragment was obtained and used for mouse immunization (S8 Fig). The immune serum successfully detected the native CsOST (53.0 kDa) in the membranous fraction, with not in the cytosolic fraction of *C*. *sinensis* (Fig 7B). In *C*. *sinensis* adults, CsOST was localized in the oral sucker and in mesenchymal tissues throughout the body, and was particularly abundant around the intestine and vitelline glands, uterus, and testes (Fig 8A, 8C, 8E, 8G and 8I).

## Discussion

*C*. *sinensis* is a bile-dwelling liver fluke that survives in the bile duct of the final host. Bile acids play an essential role in acting as physiological stimuli but can have harmful effects on *C*. *sinensis* survival [15, 43, 44]. Although there is no direct evidence concerning the toxicity of accumulated bile acids to the worm’s tissues and cells, there has been indirect evidence of toxicity or a repellant effect of bile components on parasite survival [15, 45]. Higher concentrations (>0.005%) of bile as well as scant (1 μM) LCA are unfavorable for the worms’ survival. Therefore, *C*. *sinensis* may utilize a defense mechanism against the accumulation and toxicity of bile.

Bile transporters play a crucial role in transporting bile acids in and out of tissues and cells. In mammalians, OST, BSEP, MRP2, MRP3, and MRP4 function as efflux transporters, while apical sodium-dependent bile acid transporter (ASBT) and Na^+^-taurocholate co-transporting polypeptide (NTCP) are well-known influx transporters [13, 46]. Of note, OST and ASBT orchestrate the altered homeostasis of bile acid via a FXR-FGF15-FGFR4-mediated mechanism, in which farnesoid X receptor (FXR) as well as two bile transporters regulate fibroblast growth factor 15 (FGF15) expression and in turn activate its receptor, fibroblast growth factor receptor 4 (FGFR4), to repress cytochrome P450 family 7 subfamily a member 1 (Cyp7a1) expression and bile acid synthesis [47]. To date, only two bile transporters have been identified in the parasites, MRP4 of *C*. *sinensis* (CsMRP4) [16] and BSEP of *Fasciola gigantica* [48]. Thus, the function of bile transporters in the liver fluke needs to be investigated further, in comparison with mammalian transporters.

In the present study, prior to further analysis CsOST was split into two domains, which comprised the N-terminal ordered domain (CsOST-N; Met1–Arg354) and the C-terminal disordered domain (CsOST-C; Arg355–Leu480). These domains were applied for primary sequence analysis and a threading-based 3D modeling. Separating the disordered region is a prerequisite process, as the overall quality of the 3D model can be decreased due to interference of the regions with the structural clustering process [49]. Thus, the quality of the overall model can be increased by removing a short-disordered region and by splitting a protein into two or more domains [50]. This process facilitates better templates corresponding to each domain.

CsOST-N had an ‘OSTα/TMEM184C’ domain and ‘Solute_trans_a’ domain at aa42–308, with an RXR motif at the N-terminal end and three highly conserved cysteine residues. The RXR motif serves as a retrieval signal that prevents transport of inappropriate complexes from the endoplasmic reticulum [51]. Although the function of conserved cysteine residues is unclear, it may act as a substrate binding site for interacting with OSTβ [52].

The OSTβ subunits showed a different pattern of RXR motifs compared to OSTα, and LeOSTβ did not have an LL motif. The RXR motif within the OSTβ subunits is expected to function in coordination with those in the OSTα subunits [51, 53]. In MmOSTβ, the RKK motif is required for correct membrane topology [54]. The LL motif was identified in OSTβ of mammals and in CsOST-C. The motif can be an additional determinant that prevents the cell surface expression of single subunits or misassembled complexes [51].

OST subunits are more conserved in structural features than in sequence features across different species. The observed structural similarities of CsOST-N to the OSTα subunits (61.6–63.2%) were much higher than the sequence similarities (18.0–19.9%). For CsOST-C, structural similarity to the OSTβ subunits was 23 times higher than sequence similarities (32.6–33.4% vs. 9.7–14.9%). Experimentally, the OSTα and OSTβ subunits derived from different vertebrate species formed heterodimers and functioned complementarily to each other. These findings indicate that structural similarity creates a higher probability for functional annotation than sequential similarity does [13, 20, 53]. Our results revealed a unique structural feature of LeOSTβ and insufficient structural similarity to HsOSTβ and MmOSTβ. This implies that each OST subunit is more structurally conserved around the active site across species and there is structural diversity in the OSTβ subunits. The functions of remote homologous sequences showing sequence identities of <50% should be explored at the 3D structural level rather than at the sequence level, as structural features are better evolutionary predict indices than sequence similarity [55, 56].

There is little information about OSTβ from invertebrate species [57]. However, the available information indicates that OSTβ is indispensable for heterodimerization, trafficking, and transport functions [54]. Therefore, we suggest that CsOST includes OSTα and OSTβ subunits for the following reasons. The length of CsOST (480 aa) is much longer than vertebrate OSTα subunits (326–384 aa) and is similar to a concatemer of mammalian OSTα and β subunits (468–470 aa). Second, multiple sequence comparisons of CsOST-N and -C with canonical OSTα and β subunits showed more positional conservation than overall sequence conservation. These results are consistent with previous reports of the sequence conservation of human, mouse, and skate [20, 53]. The OSTα of the little skate shows little sequence similarity to the human and mouse versions, revealing only one of the possible motifs [20]. OST of the invertebrate liver fluke, *C*. *sinensis* showed low similarity those of the vertebrates, which show identical and consecutive cysteine residues. On the other hand, Hwang et al. reported that OST sequence identities of the little skate showed 24% similarity with that of chicken (*Gallus gallus*) OST and 11% identity with human OST [53]. Interestingly, human OSTβ is considerably less similar to that of lower vertebrates, revealing different positions of all functional motifs. Third, a constructed phylogenetic tree grouped CsOST-N and CsOST-C with the OSTα and OSTβ subunits, respectively. CsOST-N was closely related to XtOSTα, supporting a functional annotation to the OSTα subunit. Fourth, CsOST-N and CsOST-C displayed higher homology with the OSTα and β subunits concerning tertiary structure rather than sequential features. Fifth, CsOST-C has a few short α-helixes, and the mostly-disordered region may fold into a stereoscopic configuration that forms a stable tertiary structure. In *Escherichia coli*, the NBD1 and NBD2 subdomains of the ABC transporter are disordered [58]. Interestingly, the disordered regions formed an ordered heterodimer when the substrates bound to the binding pocket. With these considerations, the CsOST-C was identified as CsOSTβ.

The mRNA of CsOST was more abundant in the metacercariae than in the adults of *C*. *sinensis*. This explains the finding that the developmental expression of genes in the adaptation to a bile environment are higher in the metacercariae than in adults, exemplified by the sodium/bile acid cotransporter [24] and CsMRP4 [16]. The ingested metacercariae in the duodenum might adapt to survive initial bile exposure (or bile shock) in the bile duct of the mammalian host. The *C*. *sinensis* adults utilize a high level of carbon energy sources and produce many eggs. For this purpose, the adult flukes have more glucose transporters than the metacercariae [24].

The tissue distribution of mammalian OSTs revealed that the OST acts as a bile acid transporter in the basolateral sides of ileal enterocytes and also found in the cells of liver, testis and ovary [20, 59]. In the liver flukes, several transporters were reported distributed in mesenchymal tissues, such as BSEP of adult *F*. *gigantica* [48] and CsMRP7 [21]. Distribution of these transporters suggested that the CsOST may participate in the cell-to-cell transportation and homeostasis of bile acids in the body of *C*. *sinensis*. To survive in the bile ducts, the liver flukes have to pump out bile acids from the body, since they are toxic and decrease body movements [15, 45, 60]. It is suggested that the CsOST participate in pumping out bile acids from the *C*. *sinensis* body, together with other bile acid exporters.

### Conclusion

An organic solute transporter (CsOST) of *C*. *sinensis* was identified at molecular and bioinformatics computational levels. The CsOST polypeptide had two domains, the ordered CsOST-N and the disordered CsOST-C. The CsOST-N and CsOST-C were conserved with canonical OST α- and β-subunits, and showed positional conservation rather than overall sequence conservation. Phylogenetic tree analysis supported that CsOST-N and -C could be grouped as OSTα and OSTβ subunits. The predicted 3D-structures of CsOST-N and CsOST-C revealed higher similarity to the OSTα and OSTβ subunits than did the sequence identities. With these findings, the CsOST appears to comprise N- and C-terminal domains corresponding to the OSTα and OSTβ subunits, respectively. The CsOST was localized in the mesenchymal tissues of *C*. *sinensis* body. It is suggested that the CsOST plays a role in transporting bile acids in a manner similar to mammalian OSTs.

## Supporting information

S1 Table Pairwise structural comparison between HsOSTα and MmOSTα. (DOCX)

S2 Table Pairwise structural comparison between HsOSTβ and MmOSTβ. (DOCX)

S3 Table Pairwise structural comparison between CsOST-N and the most conserved OSTα models. (DOCX)

S4 Table Pairwise structural comparison between CsOST-C and the most conserved OSTβ models. (DOCX)

S5 Table Pairwise structural comparison between LeOSTβ and the most conserved OSTβ models. (DOCX)

S1 Fig Schematic diagram of cloning full-length CsOST cDNA. An EST (CSA09963) contained 3′-UTR and poly(A)-tail (139 bp). A 5′-lost sequence was obtained by 5′-RACE. The full length of CsOST cDNA was 1725 bp and encoded 480 aa. Abbreviations are: GSP, gene-specific primer; NGSP, nested gene-specific primer; F-TA, forward primer for TA-cloning; and R-TA, reverse primer for TA-cloning.
(TIF)

S2 Fig Agarose gel electrophoresis of primary and nested PCR amplicons of 5′-RACE for CsOST cDNA. (A) An amplicon (1200 bp) by primary PCR. (B) Amplicon (1100 bp) generated by nested PCR. M, DNA size marker.
(TIF)

S3 Fig Nucleotide and deduced polypeptide sequences of CsOST cDNA. Full-length sequence (1725 bp) of CsOST cDNA encoding a polypeptide of 480 aa. 5′-UTR, 143 bp; 3′-UTR, 139 bp.
(TIF)

S4 Fig Structural comparison of LeOSTβ with other OST β-subunits. The 3D models of HsOSTβ (*red*), MmOSTβ (*blue*), and LeOSTβ (*forest green*) were superposed on each other. Pairwise comparisons were performed as follows: HsOSTβ and LeOSTβ (A), MmOSTβ and LeOSTβ (B). TM-score between two superposed structures was calculated using TM-align. Abbreviations are: HsOSTβ, *Homo sapiens* OSTβ; MmOSTβ, *Mus musculus* OSTβ; and LeOSTβ, *Leucoraja erinacea* OSTβ.
(TIF)

S5 Fig Validation of 3D model of CsOST-N. (A) Ramachandran plot shows the residues in most favored regions (87.5%), additional allowed regions (11.2%), generously allowed regions (0.7%), and disallowed regions (0.7%). Red (A, B, L), yellow (a, b, l, p), and light yellow (~a, ~b, ~l, ~p) indicate the most favored regions, allowed regions, and generously allowed regions, respectively. White shows disallowed regions. All non-glycine and non-proline residues are shown as closed black squares while glycines (non-end) are shown as closed black triangles. Generously allowed or disallowed residues are colored in red. (B) An ERRAT plot shows overall quality factor, 84.1%.
(TIF)

S6 Fig Validation of 3D model of CsOST-C. (A) Ramachandran plot shows the residues in most favored regions (78.0%), additional allowed regions (20.2%), generously allowed regions (0.9%), and disallowed regions (0.9%). Red (A, B, L), yellow (a, b, l, p), and light yellow (~a, ~b, ~l, ~p) indicate the most favored regions, allowed regions, and generously allowed regions, respectively. White shows disallowed regions. All non-glycine and non-proline residues are shown as closed black squares while glycines (non-end) are shown as closed black triangles. Generously allowed or disallowed residues are colored in red. (B) An ERRAT plot. Overall quality factor was 77.1%.
(TIF)

S7 Fig B-cell epitope prediction and hydrophilicity analysis of CsOST. A peptide of high immunogenicity and hydrophilicity was selected between amino acids 348 and 480 for recombinant CsOST production.
(TIF)

S8 Fig Induction and purification of recombinant CsOST protein. (A) rCsOST protein on 12% gradient gel stained with Coomassie blue. A predicted size was 17.5 kDa. U, uninduced total. T, induced total. S, induced soluble. PT, pass-through. W, washing. (B) Immunoblotting of purified rCsOST protein. M, protein molecular marker.
(TIF)
