# Supplementary material for: Characterization of a novel organic solute transporter homologue from Clonorchis sinensis
Source: PLoS Negl Trop Dis. 2018 Apr 27;12(4):e0006459. doi: 10.1371/journal.pntd.0006459 (PMC5942847; doi:10.1371/journal.pntd.0006459)
Supplement: S1 Table — (DOCX) [file pntd.0006459.s001.docx]

**S1 Table.** Pairwise structural comparison between HsOSTα and MmOSTα

**Top 10 models of HsOSTα**

| Model No. | No. 1 | **No. 2** | No. 3 | No. 4 | No. 5 | No. 6 | No. 7 | No. 8 | No. 9 | No. 10 |
| --- | --- | --- | --- | --- | --- | --- | --- | --- | --- | --- |
| No. 1 | 0.70 | 0.59 | 0.29 | 0.34 | 0.75 | 0.31 | 0.27 | 0.30 | 0.54 | 0.24 |
| **No. 2** | 0.61 | **0.85** | 0.31 | 0.33 | 0.60 | 0.25 | 0.31 | 0.29 | 0.55 | 0.23 |
| No. 3 | 0.32 | 0.30 | 0.32 | 0.29 | 0.33 | 0.29 | 0.30 | 0.28 | 0.29 | 0.28 |
| No. 4 | 0.21 | 0.26 | 0.34 | 0.24 | 0.23 | 0.27 | 0.23 | 0.25 | 0.25 | 0.37 |
| No. 5 | 0.69 | 0.61 | 0.27 | 0.32 | 0.79 | 0.29 | 0.29 | 0.29 | 0.54 | 0.24 |
| No. 6 | 0.30 | 0.29 | 0.36 | 0.29 | 0.30 | 0.29 | 0.25 | 0.32 | 0.28 | 0.23 |
| No. 7 | 0.26 | 0.29 | 0.27 | 0.30 | 0.28 | 0.31 | 0.76 | 0.29 | 0.28 | 0.25 |
| No. 8 | 0.29 | 0.30 | 0.31 | 0.31 | 0.28 | 0.31 | 0.26 | 0.91 | 0.26 | 0.28 |
| No. 9 | 0.22 | 0.24 | 0.25 | 0.33 | 0.24 | 0.27 | 0.26 | 0.30 | 0.27 | 0.92 |
| No. 10 | 0.45 | 0.47 | 0.36 | 0.31 | 0.45 | 0.28 | 0.27 | 0.30 | 0.41 | 0.24 |

**Top 10 models of**

**MmOSTα**

1. *Shaded* and *white* boxes show “Medium” and “low” of confidence score, respectively. The confidence score was obtained from LOMETS server.
2. Number in *red* indicates models showing the highest similarity.
