# Supplementary material for: Characterization of a novel organic solute transporter homologue from Clonorchis sinensis
Source: PLoS Negl Trop Dis. 2018 Apr 27;12(4):e0006459. doi: 10.1371/journal.pntd.0006459 (PMC5942847; doi:10.1371/journal.pntd.0006459)
Supplement: S2 Table — (DOCX) [file pntd.0006459.s002.docx]

**S2 Table.** Pairwise structural comparison between HsOSTβ and MmOSTβ

**Top 10 models of HsOSTβ**

| Model No. | No. ^^[[1]](#footnote-1)^^ | No. 2 | No. ^^[[2]](#footnote-2)^^ | No. 4 | **No. 5** | No. 6 | No. 7 | No. 8 | No. 9 | No. 10 |
| --- | --- | --- | --- | --- | --- | --- | --- | --- | --- | --- |
| No. 1 | 0.32 | 0.25 | 0.29 | 0.19 | 0.42 | 0.43 | 0.34 | 0.31 | 0.24 | 0.27 |
| No. 2 | 0.22 | 0.27 | 0.24 | 0.20 | 0.23 | 0.21 | 0.23 | 0.30 | 0.27 | 0.22 |
| No. 3 | 0.31 | 0.21 | 0.33 | 0.23 | 0.31 | 0.32 | 0.27 | 0.22 | 0.24 | 0.25 |
| **No. 4** | 0.33 | 0.18 | 0.36 | 0.17 | **0.83** | 0.47 | 0.31 | 0.23 | 0.21 | 0.24 |
| No. 5 | 0.28 | 0.21 | 0.30 | 0.20 | 0.32 | 0.45 | 0.38 | 0.17 | 0.21 | 0.18 |
| No. 6 | 0.34 | 0.19 | 0.32 | 0.19 | 0.80 | 0.46 | 0.33 | 0.22 | 0.23 | 0.23 |
| No. 7 | 0.29 | 0.19 | 0.28 | 0.17 | 0.33 | 0.39 | 0.86 | 0.17 | 0.20 | 0.20 |
| No. 8 | 0.30 | 0.30 | 0.22 | 0.26 | 0.24 | 0.28 | 0.20 | 0.41 | 0.30 | 0.24 |
| No. 9 | 0.24 | 0.27 | 0.27 | 0.25 | 0.35 | 0.38 | 0.36 | 0.16 | 0.23 | 0.24 |
| No. 10 | 0.25 | 0.22 | 0.22 | 0.25 | 0.23 | n.a. | 0.18 | 0.29 | 0.38 | 0.20 |

**Top 10 models of**

**MmOSTβ**

1. *White* boxes show “low” of confidence score. The confidence score was obtained from LOMETS server. ^2^ Number in *red* indicates models showing the highest similarity. [↑](#footnote-ref-1)
2. n.a., not available [↑](#footnote-ref-2)
