# Supplementary material for: Characterization of a novel organic solute transporter homologue from Clonorchis sinensis
Source: PLoS Negl Trop Dis. 2018 Apr 27;12(4):e0006459. doi: 10.1371/journal.pntd.0006459 (PMC5942847; doi:10.1371/journal.pntd.0006459)
Supplement: S3 Table — (DOCX) [file pntd.0006459.s003.docx]

**S3 Table.** Pairwise structural comparison between CsOST-N and the most conserved OSTα models

**HsOSTα**  **MmOSTα**

| Model No. | **No. 2** | **No. 2** |
| --- | --- | --- |
| **No. 1** | **0.62** | **0.63** |
| No. 2 | 0.19 | 0.18 |
| No. 3 | 0.59 | 0.60 |
| No. 4 | 0.30 | 0.29 |
| No. 5 | 0.30 | 0.31 |
| No. 6 | 0.60 | 0.61 |
| No. 7 | 0.58 | 0.57 |
| No. 8 | 0.30 | 0.29 |
| No. 9 | 0.21 | 0.22 |
| No. 10 | 0.19 | 0.19 |

**Top 10 models of**

**CsOST-N**

1. *Shaded* and *white* boxes show “Medium” and “low” of confidence score, respectively. The confidence score was obtained from LOMETS server.
2. Number in *red* indicates models showing the highest similarity.
