# Supplementary material for: Characterization of a novel organic solute transporter homologue from Clonorchis sinensis
Source: PLoS Negl Trop Dis. 2018 Apr 27;12(4):e0006459. doi: 10.1371/journal.pntd.0006459 (PMC5942847; doi:10.1371/journal.pntd.0006459)
Supplement: S4 Table — (DOCX) [file pntd.0006459.s004.docx]

**S4 Table.** Pairwise structural comparison between CsOST-C and the most conserved OSTβ models

**HsOSTβ MmOSTβ**

| Model No. | **No. 5** | **No. 4** |
| --- | --- | --- |
| No. 1 | 0.32 | 0.3^^[[1]](#footnote-1)^^ |
| No. 2 | 0.26 | 0.26 |
| No. ^^[[2]](#footnote-2)^^ | n.a. | n.a. |
| **No. 4** | **0.33** | **0.34** |
| No. 5 | 0.22 | 0.22 |
| No. 6 | 0.28 | 0.29 |
| No. 7 | 0.29 | 0.32 |
| No. 8 | 0.27 | 0.28 |
| No. 9 | 0.26 | 0.23 |
| No. 10 | 0.26 | 0.26 |

**Top 10 models of**

**CsOST-C**

1. *White* boxes show “low” of confidence score. The confidence score was obtained from LOMETS server. ^2^ Number in *red* indicates models showing the highest similarity. [↑](#footnote-ref-1)
2. n.a., not available [↑](#footnote-ref-2)
