# Supplementary material for: Characterization of a novel organic solute transporter homologue from Clonorchis sinensis
Source: PLoS Negl Trop Dis. 2018 Apr 27;12(4):e0006459. doi: 10.1371/journal.pntd.0006459 (PMC5942847; doi:10.1371/journal.pntd.0006459)
Supplement: S5 Table — (DOCX) [file pntd.0006459.s005.docx]

**S5 Table.** Pairwise structural comparison between LeOSTβ and the most conserved OSTβ models

**HsOSTβ** **MmOSTβ**

| Model No. | **No. 5** | **No. 4** |
| --- | --- | --- |
| No. 1 | 0.31 | 0.32 |
| No. 2 | 0.32 | 0.32 |
| No. 3 | 0.30 | 0.30 |
| **No. 4** | **0.41** | **0.41** |
| No. 5 | 0.38 | 0.38 |
| No. 6 | n.a. | n.a. |
| No. 7 | n.a. | n.a. |
| No. 8 | n.a. | n.a. |
| No. 9 | n.a. | n.a. |
| No. 10 | n.a. | n.a. |

**Top 10 models of**

**LeOSTβ**

^1^ *White* boxes show “low” of confidence score. The confidence score was obtained from LOMETS server. ^2^ Number in *red* indicates models showing the highest similarity. ^3^ n.a., not available
