# Supplementary figures and images for: Characterization of a novel organic solute transporter homologue from Clonorchis sinensis
Source: PLoS Negl Trop Dis. 2018 Apr 27;12(4):e0006459. doi: 10.1371/journal.pntd.0006459 (PMC5942847; doi:10.1371/journal.pntd.0006459)

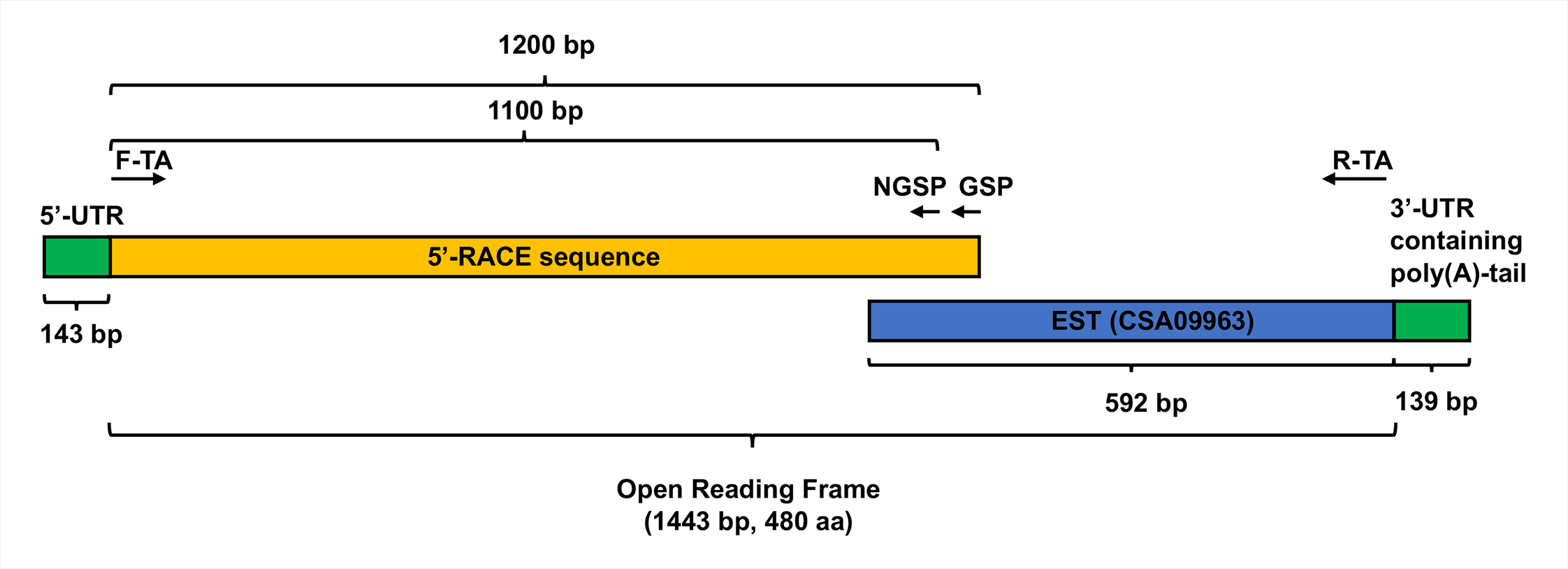

Supplement: S1 Fig — An EST (CSA09963) contained 3′-UTR and poly(A)-tail (139 bp). A 5′-lost sequence was obtained by 5′-RACE. The full length of CsOST cDNA was 1725 bp and encoded 480 aa. Abbreviations are: GSP, gene-specific primer; NGSP, nested gene-specific primer; F-TA, forward primer for TA-cloning; and R-TA, reverse primer for TA-cloning. (TIF) [file pntd.0006459.s006.tif]

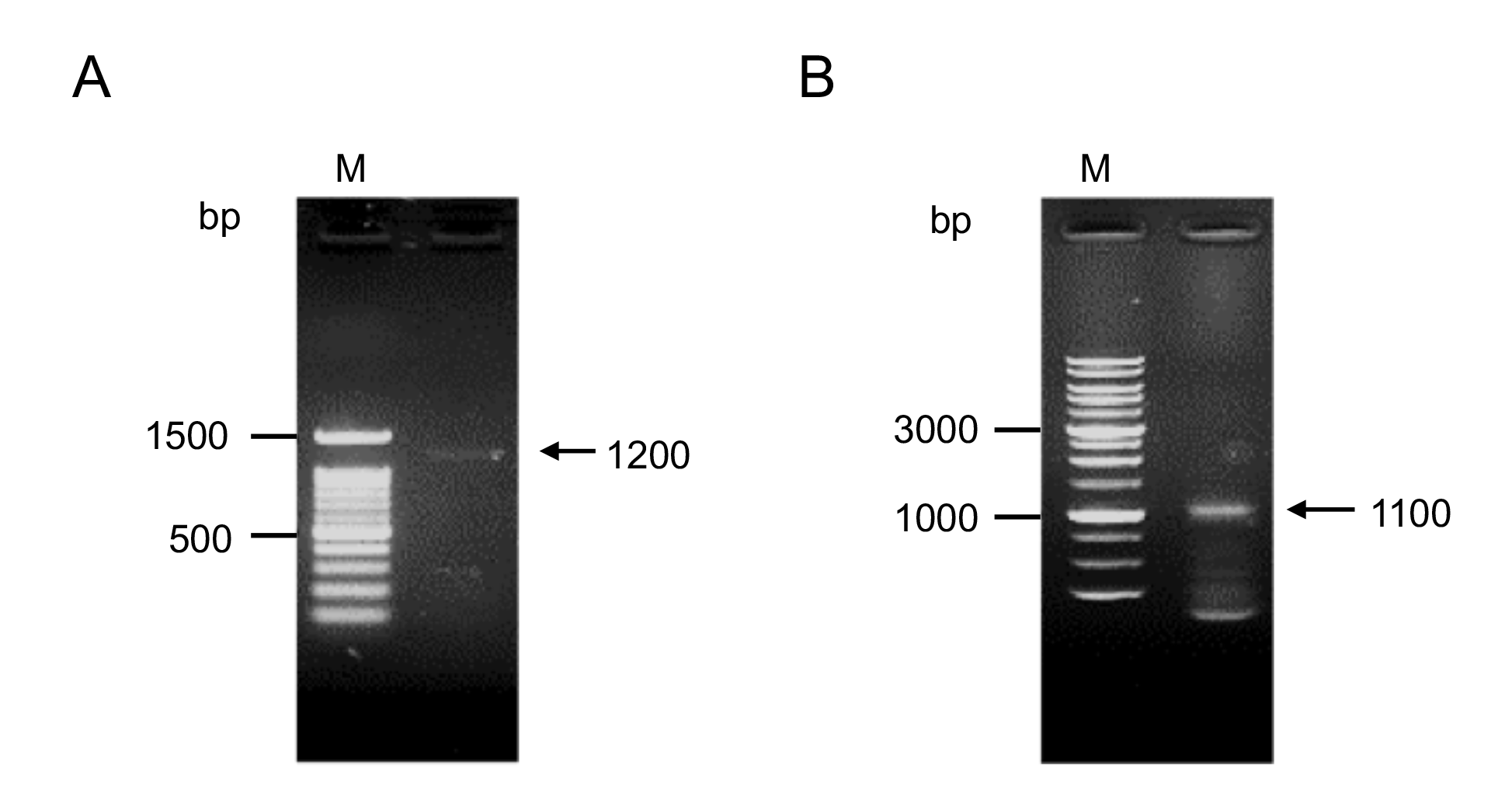

Supplement: S2 Fig — (A) An amplicon (1200 bp) by primary PCR. (B) Amplicon (1100 bp) generated by nested PCR. M, DNA size marker. (TIF) [file pntd.0006459.s007.tif]

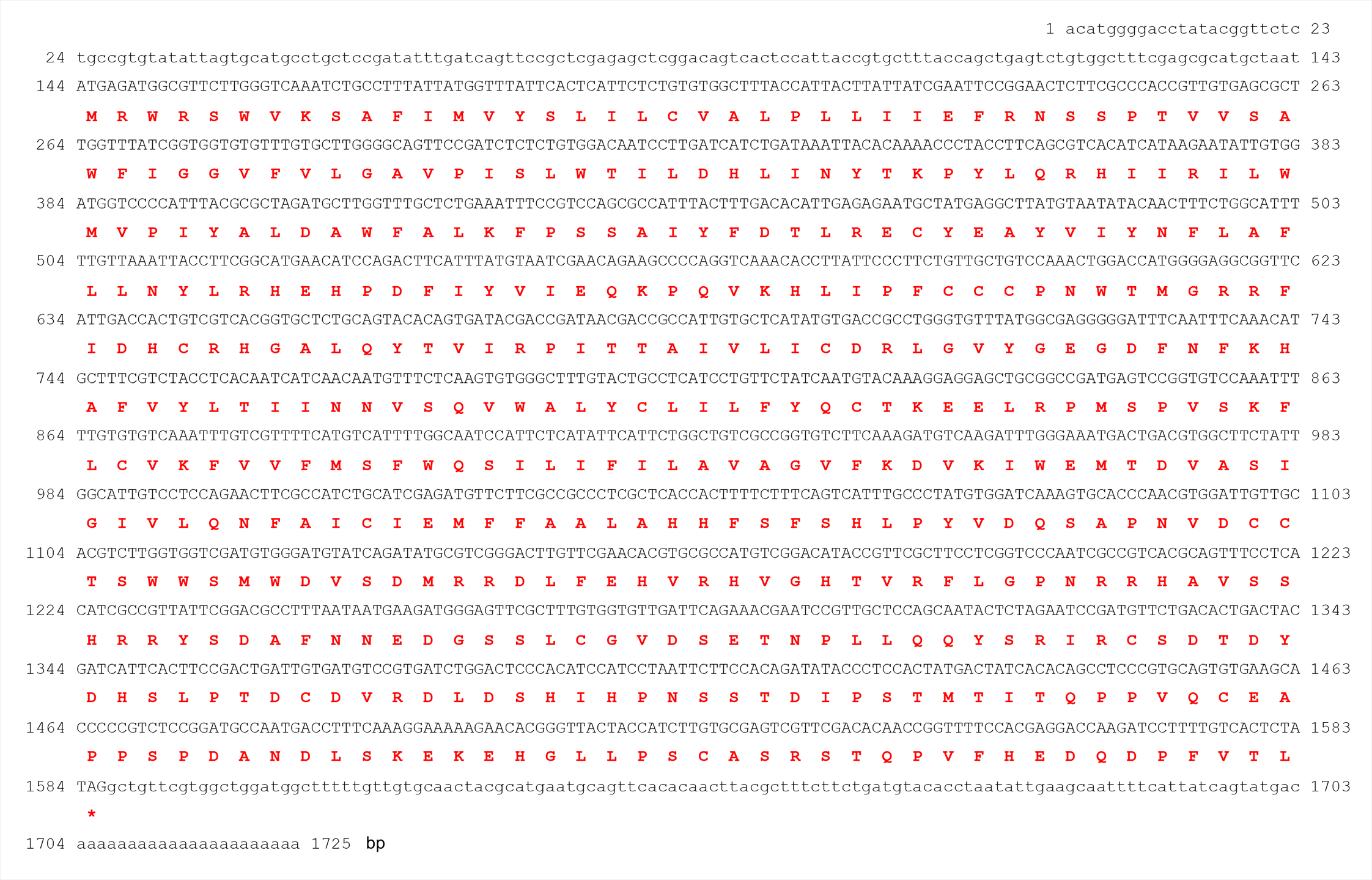

Supplement: S3 Fig — Full-length sequence (1725 bp) of CsOST cDNA encoding a polypeptide of 480 aa. 5′-UTR, 143 bp; 3′-UTR, 139 bp. (TIF) [file pntd.0006459.s008.tif]

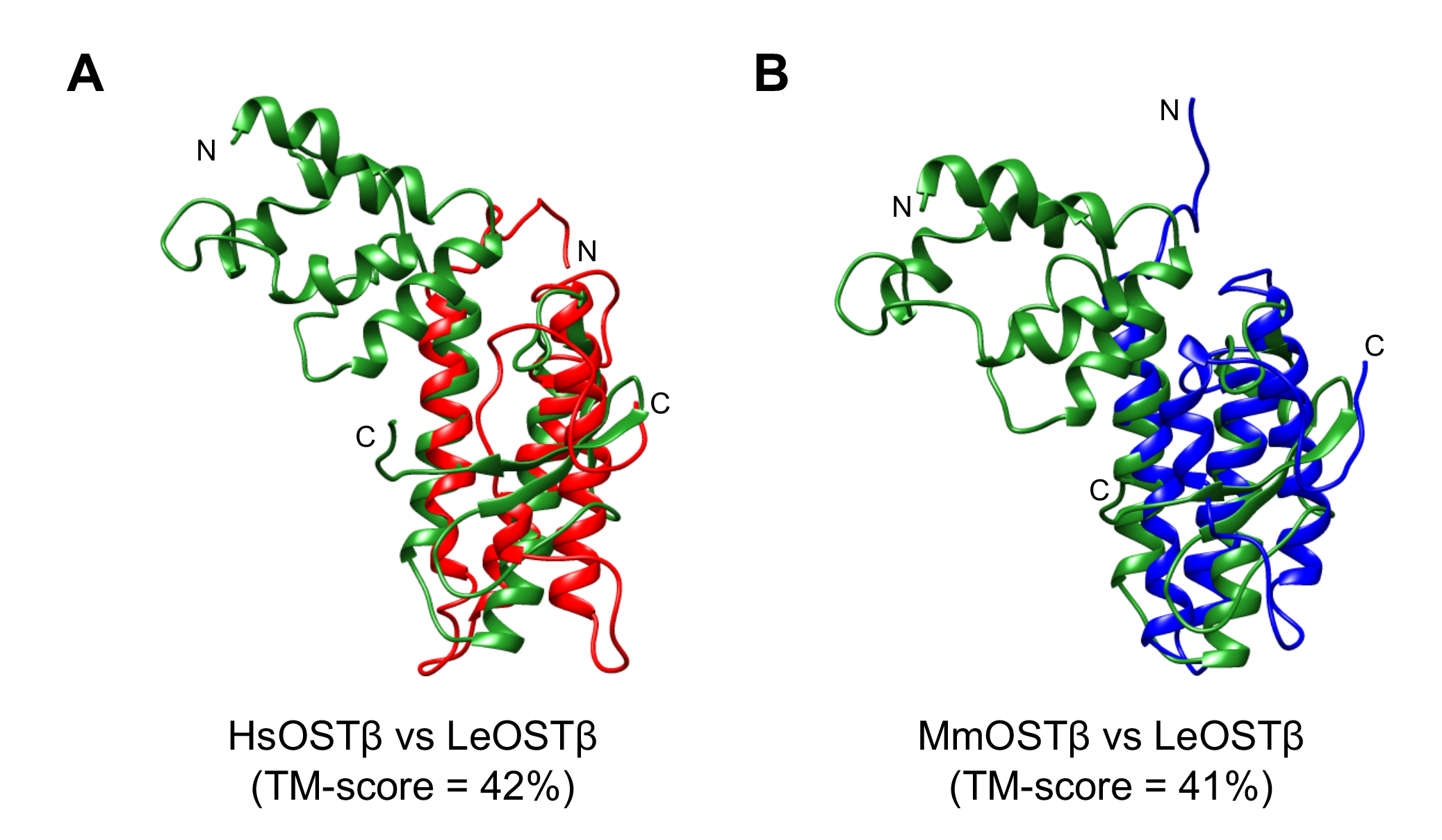

Supplement: S4 Fig — The 3D models of HsOSTβ (red), MmOSTβ (blue), and LeOSTβ (forest green) were superposed on each other. Pairwise comparisons were performed as follows: HsOSTβ and LeOSTβ (A), MmOSTβ and LeOSTβ (B). TM-score between two superposed structures was calculated using TM-align. Abbreviations are: HsOSTβ, Homo sapiens OSTβ; MmOSTβ, Mus musculus OSTβ; and LeOSTβ, Leucoraja erinacea OSTβ. (TIF) [file pntd.0006459.s009.tif]

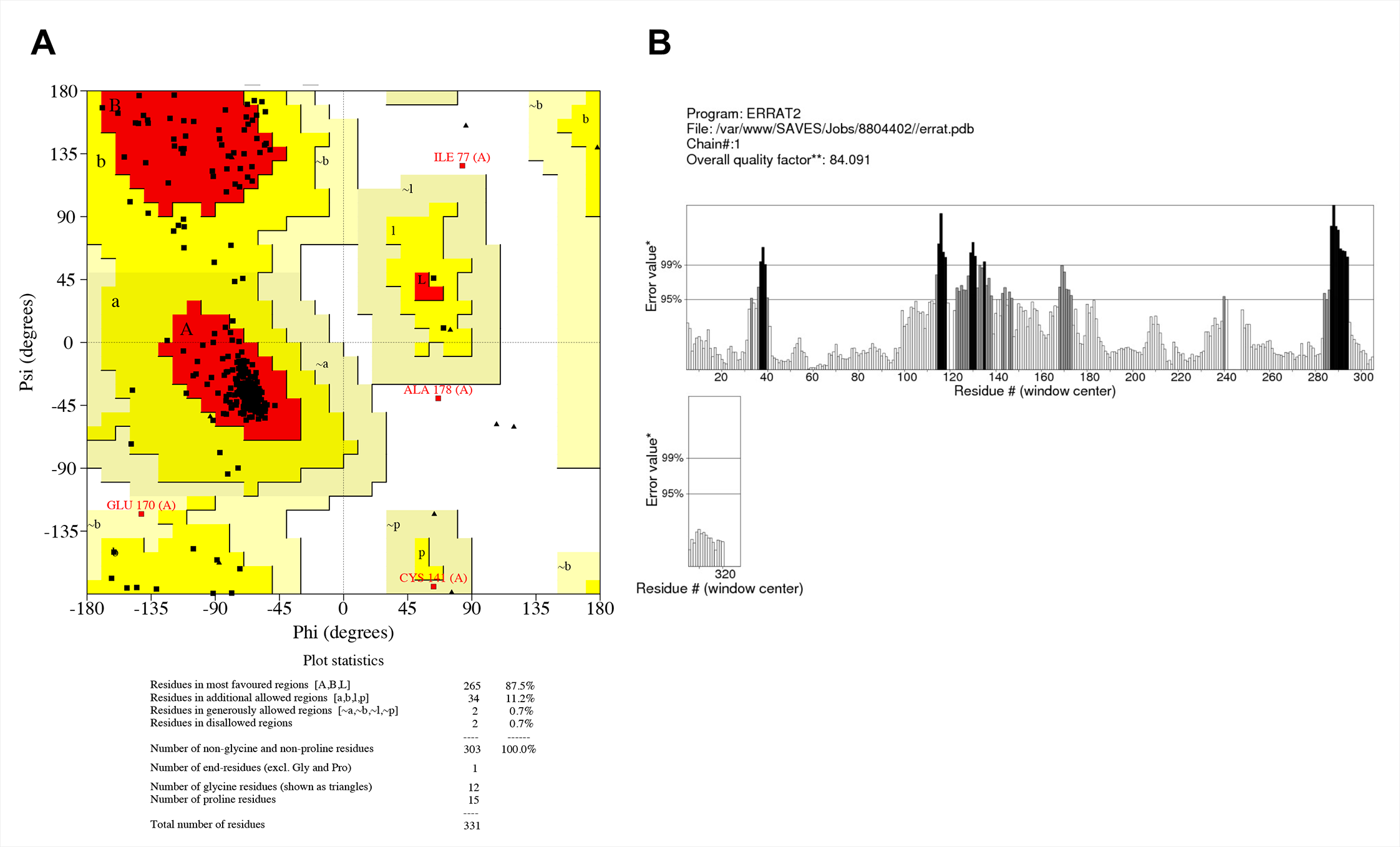

Supplement: S5 Fig — (A) Ramachandran plot shows the residues in most favored regions (87.5%), additional allowed regions (11.2%), generously allowed regions (0.7%), and disallowed regions (0.7%). Red (A, B, L), yellow (a, b, l, p), and light yellow (~a, ~b, ~l, ~p) indicate the most favored regions, allowed regions, and generously allowed regions, respectively. White shows disallowed regions. All non-glycine and non-proline residues are shown as closed black squares while glycines (non-end) are shown as closed black triangles. Generously allowed or disallowed residues are colored in red. (B) An ERRAT plot shows overall quality factor, 84.1%. (TIF) [file pntd.0006459.s010.tif]

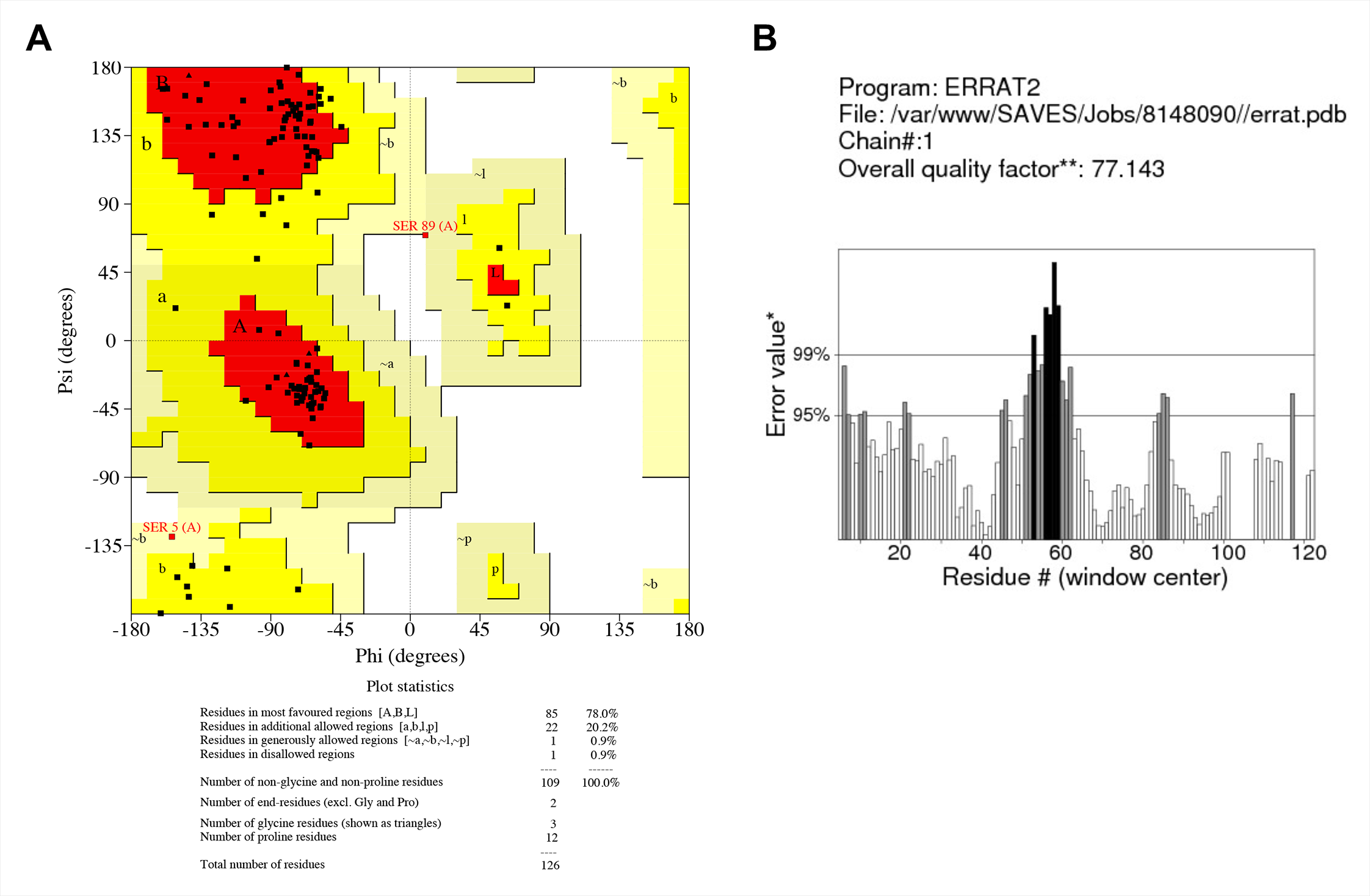

Supplement: S6 Fig — (A) Ramachandran plot shows the residues in most favored regions (78.0%), additional allowed regions (20.2%), generously allowed regions (0.9%), and disallowed regions (0.9%). Red (A, B, L), yellow (a, b, l, p), and light yellow (~a, ~b, ~l, ~p) indicate the most favored regions, allowed regions, and generously allowed regions, respectively. White shows disallowed regions. All non-glycine and non-proline residues are shown as closed black squares while glycines (non-end) are shown as closed black triangles. Generously allowed or disallowed residues are colored in red. (B) An ERRAT plot. Overall quality factor was 77.1%. (TIF) [file pntd.0006459.s011.tif]

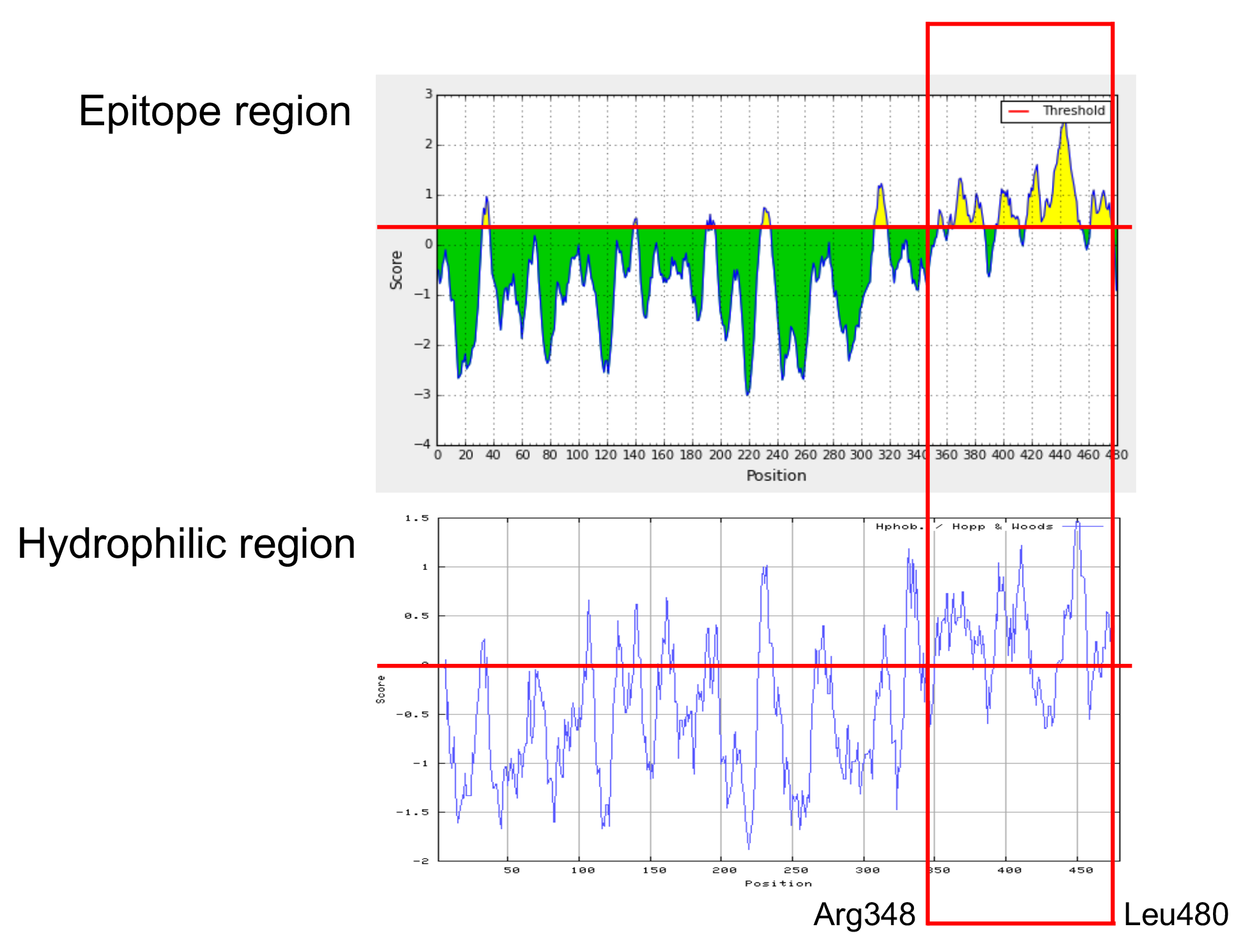

Supplement: S7 Fig — A peptide of high immunogenicity and hydrophilicity was selected between amino acids 348 and 480 for recombinant CsOST production. (TIF) [file pntd.0006459.s012.tif]

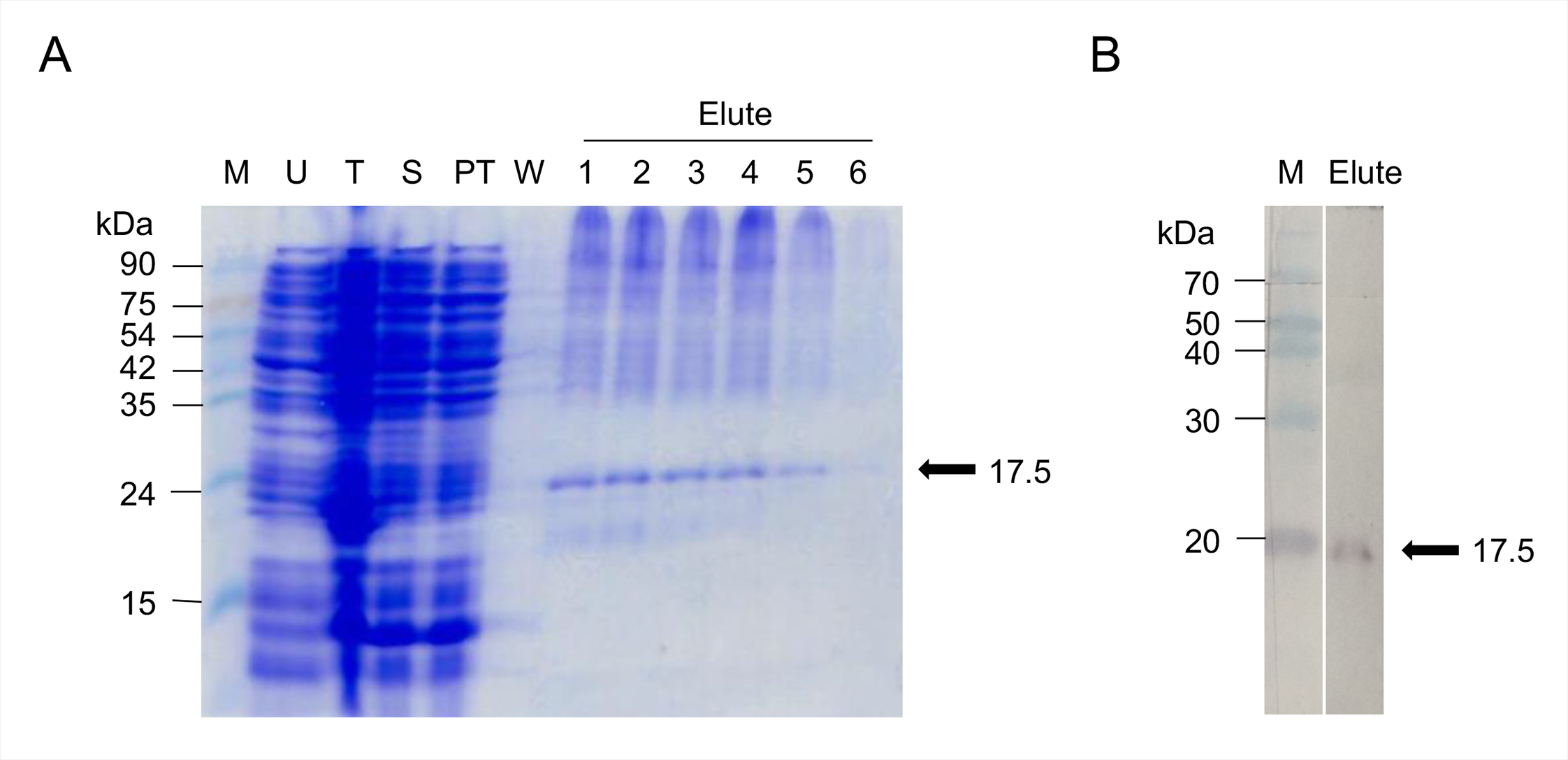

Supplement: S8 Fig — (A) rCsOST protein on 12% gradient gel stained with Coomassie blue. A predicted size was 17.5 kDa. U, uninduced total. T, induced total. S, induced soluble. PT, pass-through. W, washing. (B) Immunoblotting of purified rCsOST protein. M, protein molecular marker. (TIF) [file pntd.0006459.s013.tif]
